# Decrements in lung function and respiratory abnormalities associated with exposure to diacetyl and 2,3-pentanedione in coffee production workers

**DOI:** 10.3389/fpubh.2022.966374

**Published:** 2022-08-12

**Authors:** Mohammed Abbas Virji, Ethan D. Fechter-Leggett, Caroline P. Groth, Xiaoming Liang, Brie H. Blackley, Marcia L. Stanton, Ryan F. LeBouf, R. Reid Harvey, Rachel L. Bailey, Kristin J. Cummings, Jean M. Cox-Ganser

**Affiliations:** ^1^Respiratory Health Division, National Institute for Occupational Safety and Health, Centers for Disease Control and Prevention, Morgantown, WV, United States; ^2^Department of Epidemiology and Biostatistics, West Virginia University School of Public Health, Morgantown, WV, United States

**Keywords:** coffee production, diacetyl, 2, 3-pentanedione, spirometry, impulse oscillometry, peak exposures, restrictive pattern, small airways

## Abstract

Coffee production workers are exposed to complex mixtures of gases, dust, and vapors, including the known respiratory toxins, diacetyl, and 2,3-pentanedione, which occur naturally during coffee roasting and are also present in flavorings used to flavor coffee. This study evaluated the associations of these two α-diketones with lung function measures in coffee production workers. Workers completed questionnaires, and their lung function was assessed by spirometry and impulse oscillometry (IOS). Personal exposures to diacetyl, 2,3-pentanedione, and their sum (Sum_DA+PD_) were assigned to participants, and metrics of the highest 95th percentile (P95), cumulative, and average exposure were calculated. Linear and logistic regression models for continuous and binary/polytomous outcomes, respectively, were used to explore exposure-response relationships adjusting for age, body mass index, tenure, height, sex, smoking status, race, or allergic status. Decrements in percent predicted forced expiratory volume in 1 second (ppFEV_1_) and forced vital capacity (ppFVC) were associated with the highest-P95 exposures to 2,3-pentanedione and Sum_DA+PD_. Among flavoring workers, larger decrements in ppFEV_1_ and ppFVC were associated with highest-P95 exposures to diacetyl, 2,3-pentanedione, and Sum_DA+PD_. Abnormal FEV_1_, FVC, and restrictive spirometric patterns were associated with the highest-P95, cumulative, and average exposures for all α-diketone metrics; some of these associations were also present among flavoring and non-flavoring workers. The combined category of small and peripheral airways plus small and large airways abnormalities on IOS had elevated odds for highest-P95 exposure to α-diketones. These results may be affected by the small sample size, few cases of abnormal spirometry, and the healthy worker effect. Associations between lung function abnormalities and exposure to α-diketones suggest it may be prudent to consider exposure controls in both flavoring and non-flavoring settings.

## Introduction

Coffee production workers are exposed to complex mixtures of gases, dust, and vapors such as carbon monoxide, carbon dioxide, coffee dust, green-bean allergens, and α-diketones, including 2,3-butanedione (diacetyl—a commonly used synonym) and 2,3-pentanedione (acetyl propionyl), and other volatile organic compounds (VOCs), including acetoin (1–6). Adverse respiratory health outcomes such as respiratory symptoms, pulmonary function abnormalities, asthma, and obliterative bronchiolitis (OB) can occur among exposed coffee production workers (7–9). OB is a rare, irreversible lung disease characterized by inflammation and bronchiolar wall fibrosis, leading to luminal narrowing of the small airways (i.e., bronchioles) and obliteration that obstructs airflow (10, 11). OB has been found among workers exposed to diacetyl present in flavoring chemicals used in flavoring manufacturing and a variety of food processing industries, including coffee production (11–14). Additionally, exposure to diacetyl is associated with lung function abnormalities including fixed obstructive, restrictive, and mixed patterns on spirometry, as well as longitudinal declines in forced expiratory volume in 1 s (FEV_1_), forced vital capacity (FVC), and FEV_1_/FVC ratio, with or without respiratory symptoms (11–13, 15–21). Symptoms can include cough, shortness of breath on exertion, or wheezing, which do not improve away from work (11). Respiratory health risk from exposure to 2,3-pentanedione has not been evaluated in epidemiologic studies, but animal studies report similar toxicity to that of diacetyl (22, 23). In 2016, the National Institute for Occupational Safety and Health (NIOSH) established recommended exposure limits (RELs) of 5 parts per billion (ppb) and 9.3 ppb, and short-term exposure limits (STEL) of 25 ppb and 31 ppb for diacetyl and 2,3-pentanedione, respectively (11).

In previous studies of microwave popcorn workers exposed to flavoring chemicals, decrements in lung function, i.e., lower FEV_1_ and FEV_1_/FVC ratio, were associated with average and cumulative diacetyl exposure (11). At one of these microwave popcorn production facilities, higher cumulative diacetyl exposure (quartiles) was significantly associated with a higher prevalence of airway obstruction (24). Conversely, in a study of flavoring manufacturing workers, higher duration of work in a diacetyl plant was associated with better lung function, i.e., higher percent predicted FEV_1_ (ppFEV_1_), which was attributed to potential exposure misclassification, healthy worker effect, and not accounting for the effect of peak exposure (17). Other studies have reported associations of adverse respiratory health outcomes with proxies of diacetyl exposure such as tenure or type of production activity (13, 18, 25, 26). Although metrics of peak exposure have not been available to evaluate exposure-response relationships, peak exposures to diacetyl have been documented in settings where OB cases have occurred, including the microwave popcorn industry, a flavoring manufacturing facility, and a coffee production facility and may have contributed to disease development with relatively lower average exposures (2, 13, 17, 27).

The goal of this study was to explore exposure–response relationships in coffee production for various lung function measures with a range of exposure metrics including highest, average, and cumulative exposure intensity for individual and combined α-diketone exposures.

## Methods

### Study design and population

Cross-sectional exposure and health surveys were conducted from 2016 to 2017 in response to health hazard evaluation (HHE) requests received by NIOSH from 17 small- to medium-sized coffee facilities. The plants ranged in size, the number of workers employed, production volume, the type of coffee produced, including flavored, non-flavored, or both, and other characteristics previously described (1). All current employees were invited to participate in the exposure and health assessment surveys, and written informed consent was obtained from each study participant. After the HHE investigations were completed, data from the 17 investigations were pooled to increase the sample size to evaluate exposure–response relationships that might otherwise not have been evident within each facility. The study protocol for the secondary analysis of the pooled data was approved by the NIOSH Institutional Review Board (IRB).

### Medical evaluations and health outcome measures

A combination of methods was used to characterize the health outcomes, described in detail elsewhere (7). Briefly, a standardized questionnaire was administered that included questions on demographics, symptoms and diagnoses, smoking history, work history, and exposure modules. Spirometry testing was conducted following the American Thoracic Society guidelines, and measurements were compared to their lower limit of normal (LLN) values (28, 29). Obstruction was defined as FEV_1_/FVC ratio less than the LLN with normal FVC; restrictive pattern as FVC less than the LLN with normal FEV_1_/FVC ratio; and mixed obstruction and restrictive pattern as having FVC and FEV_1_/FVC ratio less than their respective LLNs (30). Previously, obstruction was defined as FEV_1_ and FEV_1_/FVC ratio less than their respective LLNs (7). For data analysis, mixed pattern (*n* = 2) was combined with restrictive pattern (hereafter referred to as restrictive pattern) because of the small sample size; mixed pattern as indicated by spirometry may indicate a combination of physiological restriction and obstruction, and restrictive spirometry pattern may indicate physiological restriction or be caused by a physiological obstruction such as from air-trapping and small airway disease (15).

Impulse oscillometry (IOS) was performed using the CareFusion IOS system (CareFusion, Hochberg, Germany) according to the manufacturer's instructions. Details of the IOS parameters are described in Supplementary methods. Briefly, IOS parameters include (1) resistance at an oscillation frequency of 5 Hz (total resistance – small and large airways) and 20 Hz (proximal resistance – large airways) (R_5_, R_20_); (2) frequency dependence of resistance obtained as the difference between R_5_ and R_20_ (R_5−20_); (3) reactance at 5 Hz (distal capacitance – peripheral) (X_5_); (4) resonant frequency (*f*_res_); and 5) reactance area (AX) calculated as the area under the reactance curve from 5 Hz to *f*_res_ (31). Percent difference R_5_-R_20_ (DR_5−20_) is calculated as ((R_5_-R_20_)/R_20_)^*^100%; ppR_5_ is the percent predicted R_5_. Small airways and peripheral abnormality was defined as (DR_5−20_ ≥ 30%) or (ppR_5_ ≥140%, [X_5_ predicted – X_5_ measured] ≥ 0.15 kPa/(L/s) and DR_5−20_ ≥ 30%), or (ppR_5_ <140% and [X_5_ predicted – X_5_ measured] ≥ 0.15 kPa/(L/s)); large and central airways abnormality was defined as (ppR_5_ ≥ 140%, [X_5_ predicted – X_5_ measured] <0.15 kPa/(L/s) and DR_5−20_ <30%); small and large airway abnormality was defined as (ppR_5_ ≥ 140%, [X_5_ predicted – X_5_ measured] ≥ 0.15 kPa/(L/s) and DR_5−20_ < 30%); and any IOS abnormality was defined as ppR_5_ ≥ 140% or [X_5_ predicted – X_5_ measured] ≥ 0.15 kPa/(L/s) (32, 33). For data analysis, small airways and peripheral abnormality were combined with small and large airway abnormality (hereafter referred to as small airways) to emphasize any abnormality involving small airways.

### Job/task exposure matrices and exposure assignment

**Figure 1:**
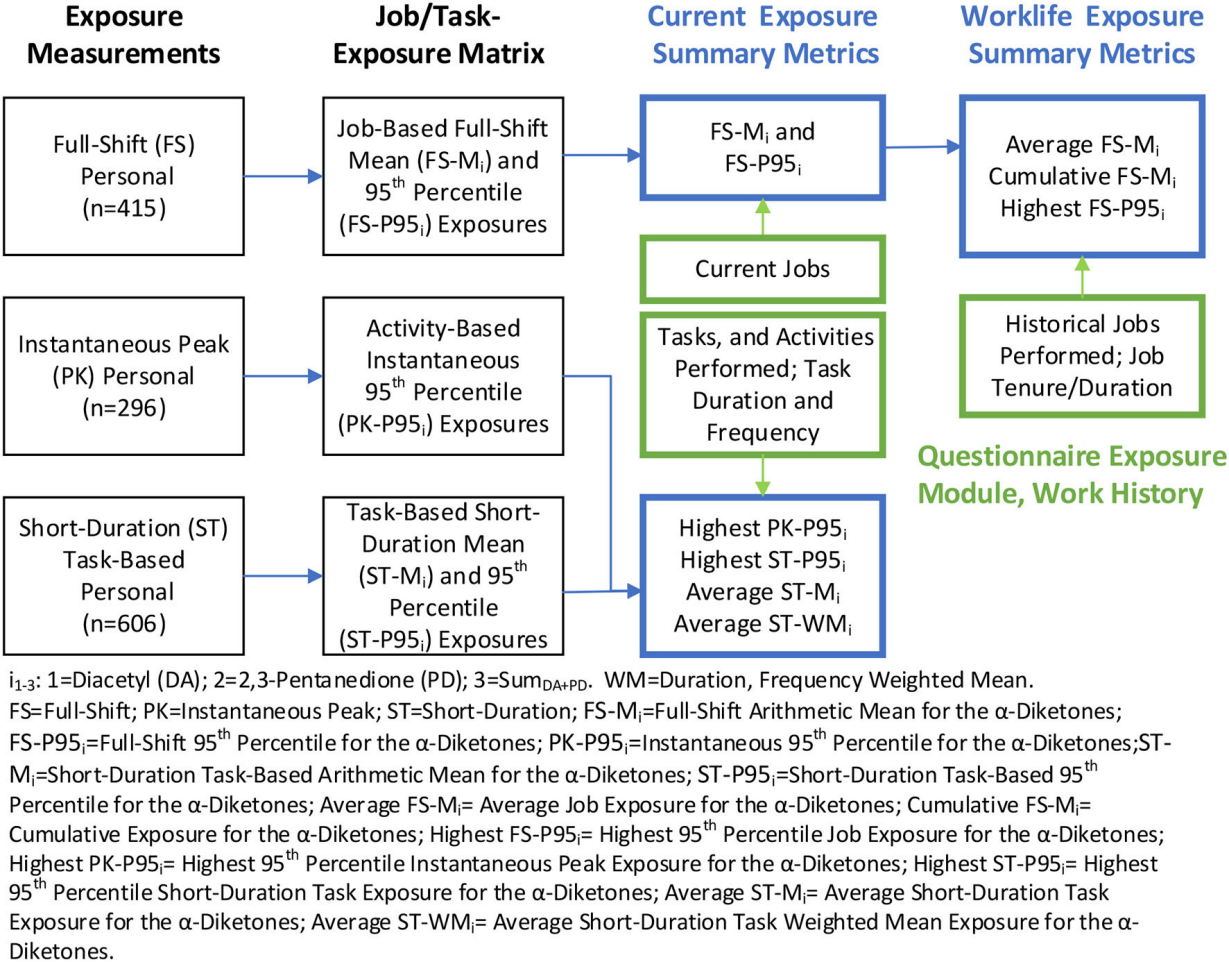
Creation of job/task-exposure matrix and summary exposure metrics. Blue text boxes include metrics used in the epidemiologic analyses; green text boxes include information gathered from the questionnaire; black text boxes include exposure data and the summary metrics in the JEM/TEM.

Job- and task-exposure matrices (JEM/TEM) were created using personal full-shift, short-duration task, and instantaneous activity measurements collected at 17 coffee facilities as outlined in Figure 1 and described in detail in Supplementary materials. Briefly, exposure measurements were summarized overall, as well as stratified by facility, facility size category, and flavoring status, using a Bayesian approach that accounts for censored data and repeated measurements (1). The mean and standard deviation of the log-transformed exposures were obtained from these models and were used to calculate the minimum variance unbiased estimator (MVUE) of the arithmetic means (AM) (34). The 95th percentile (P95) was calculated as (geometric mean) × (geometric standard deviation)^1.645^. The JEM included the AM and P95 for diacetyl, 2,3-pentanedione, and the sum of the two α-diketones (Sum_DA+PD_) for all jobs, overall and stratified by the categories as depicted in the second column of Figure 1. The JEM included estimates based on current exposures only, as historical exposure data have not previously been collected at coffee production facilities.

The AM and P95 from the JEM were then assigned to all the jobs reported by each participant in their work history. Past jobs at any facility were assigned current job exposure estimates because historical exposures were expected to be similar to current exposures; facility owners reported that no systematic changes occurred in the past that may impact exposures. The AM and P95 of the most recent job were labeled as the average and highest “current exposure” metrics in parts per billion (ppb—depicted in the third column, first row of Figure 1). The profiles of AM and P95 from multiple jobs held by workers were summarized to obtain worklife (tenure in coffee or flavoring-related work) average, cumulative (AM × duration in ppb-years) and highest (P95) summary exposure metrics for each worker (depicted in the fourth column, the first row of Figure 1). The P95 metric represents the upper tail of exposure distribution, likely resulting from high exposure tasks within a job, non-routine maintenance activity, or unplanned upset conditions (35). Thus, the highest P95 metric may be considered a surrogate of peak exposure based on full-shift measurements.

A similar approach was used to construct the TEM and assign exposures to participants as described in Supplementary materials.

### Statistical analysis

Statistical analyses were conducted using SAS software version 9.4 and JMP software version 15 (SAS Institute, Inc., Cary, NC), and plots were prepared in SigmaPlot 14.0 (Systat Software Inc., San Jose, CA). Summary statistics and correlation coefficients (Spearman rho—ρ) were calculated, and distributions were explored *via* histograms and probability plots for the various exposure metrics and continuous lung function measurements. Multiple linear regression was used to fit models for continuous outcomes, and logistic regression was used to model binary and polytomous outcomes for measures of IOS and spirometry. Metrics of highest, average, and cumulative exposure to diacetyl, 2,3-pentanedione, and Sum_DA+PD_ were fit in separate models due to collinearity among exposure metrics. Models were adjusted for age, sex, race, body mass index (BMI), height, smoking status, allergic status, or tenure as documented in the footnotes of each table. Interactions between exposure variables and tenure or flavoring status were explored to evaluate whether tenure or flavoring status modified the effect of exposure on the health outcome. Covariates were included regardless of statistical significance (11), even though they are partially accounted for in the spirometric and IOS prediction equations, to account for any potential variations from the reference population. Some covariates were dichotomized or excluded from the logistic regression models when they caused a complete or quasi-complete separation of data points (36). Odds ratios (OR) with corresponding 95% confidence intervals (CI) were obtained for the categorical outcomes, and parameter estimates (slope, β) and their 95% CI were obtained for continuous outcomes. To improve interpretability, the model parameter estimates were multiplied by 10, so the effect estimates (i.e., slope and OR) are per 10 ppb of exposure. To evaluate the performance of various exposure metrics for given outcome variables, measures of precision (parameter estimate/standard error—β/SE) and model fit (Akaike information criterion—AIC) were used to compare models (37).

## Results

### Demographics, exposure, and health distributions

Participation in the health and exposure assessments ranged from 16 to 100% and 18 to 100%, respectively, by facility. A total of 384 (58%) workers completed the health assessments, and 227 (34%) workers participated in the exposure assessment survey. As reported previously, a majority of the study population was men (59%), white (59%), and never smokers (57%), with a median age of 35 years (range: 18–72 years), median tenure across all coffee and flavoring jobs of 3.8 years (range: <1–34 years), and 35% with a BMI of >30 (7). Among all workers, 10.1% (37/367) had any abnormal spirometry, with nine workers having a restrictive pattern, 26 with obstruction, and two with a mixed pattern. The prevalence of any abnormal IOS was 27.5% (104/378), with 28 having abnormalities in the large and central airways, 18 in the small and large airways, and 58 in the small and peripheral airways. Self-reported physician-diagnosed current asthma was reported by 38/384 (9.9%) workers.

Histograms of the worklife exposure metrics show distributions with right skew; lung function parameters appear to follow a normal distribution (Supplementary Figure 1). Histograms of highest-P95 and average exposure for diacetyl and 2,3-pentanedione show most workers' assigned exposures were above the relevant RELs. The ranges of correlations within and across the different types of metrics are displayed as a heatmap in Supplementary Figure 2. Duration of exposure was negatively correlated with all exposure metrics except cumulative exposure. Metrics based on instantaneous activity were not correlated with any other metrics. Short-duration peak exposures were poorly correlated with other metrics, as were metrics of cumulative exposure. There were some moderate and some high correlations among worklife exposure metrics. Scatterplots of diacetyl vs. 2,3-pentanedione for highest-P95, cumulative, and average exposure show a high correlation within exposure metrics (Supplementary Figure 3).

### Bivariate summaries

Table 1 summarizes demographics, symptoms, spirometry, IOS, and exposure values by categories of health outcome and having ever or never held a flavoring job (hereafter referred to as flavoring). Workers who reported current asthma had the highest prevalence of all but one respiratory symptom, followed by those with abnormal spirometry; the latter group had the highest prevalence of more severe shortness of breath. Workers with abnormal spirometry or IOS had the highest exposures across all metrics, while those with asthma had similar or lower exposures compared to the group with no disease or abnormalities. The group with abnormal spirometry was mostly men and white, with the highest prevalence of flavoring jobs. Those with abnormal IOS had the highest prevalence of BMI >30 and the lowest prevalence of white race. Flavoring workers had a higher prevalence of abnormal spirometry and IOS as well as higher exposures across all metrics compared to those who never held a flavoring job; symptoms, spirometric parameters, and demographics were similar between the flavoring and non-flavoring groups.

**Table 1:** Summary of respiratory health outcome and exposure characteristics by categories of health and flavoring status.

	**Health status**				**Flavoring status**	
**Health/exposure measure**	**Any abnormal spirometry** ***N* = 37**	**Any abnormal IOS** ***N* = 104**	**Current asthma *N* = 38**	**No disease/** **abnormality** ***N* = 225**	**Flavoring job anywhere *N* = 71**	**Non-flavoring job *N* = 313**
**Symptoms in the past 12 months:** ***N*** **(%)**						
Upper respiratory	25 (67.6)	65 (62.5)	33 (86.8)	146 (64.9)	44 (62)	208 (66.5)
Lower respiratory	26 (70.3)	58 (55.8)	37 (97.4)	84 (37.3)	28 (39.4)	151 (48.2)
Breathing trouble	14 (37.8)	22 (21.2)	28 (73.7)	34 (15.1)	11 (15.5)	68 (21.7)
Cough	7 (18.9)	15 (14.4)	10 (26.3)	14 (6.2)	10 (14.1)	30 (9.6)
Wheeze	19 (51.4)	29 (27.9)	29 (76.3)	37 (16.4)	17 (23.9)	77 (24.6)
Chest tightness	4 (10.8)	14 (13.5)	19 (50)	27 (12.0)	7 (9.9)	46 (14.7)
Shortness of breath (SOB)	8 (21.6)	24 (23.1)	19 (50)	20 (8.9)	12 (16.9)	47 (15)
Severe SOB	4 (50.0)	10 (41.7)	9 (47.4)	4 (20.0)	4 (33.3)	14 (29.8)
Awoken with SOB	5 (13.5)	9 (8.7)	12 (31.6)	10 (4.4)	6 (8.5)	22 (7)
Asthma attack	8 (21.6)	9 (8.7)	24 (63.2)	1 (0.4)	6 (8.5)	20 (6.4)
**Lung function: Mean (Std) or** ***N*** **(%)**						
ppFEV_1_	82.9 (15.17)	96.2 (15.2)	96.2 (11.4)	106.0 (11.5)	101.3 (14.4)	102.6 (13.2)
ppFVC	98.9 (16.6)	99.8 (10.4)	103.8 (10.7)	105.4 (12.1)	103.1 (13.5)	103.8 (12)
ppFEV_1_/FVC Ratio	84.7 (16.2)	96.1 (11.6)	92.3 (7.5)	100.4 (5.6)	98.1 (9.6)	98.6 (8.5)
Abnormal spirometry	37 (100)	18 (18.0)	9 (25.7)	0 (–)	11 (16.4)	26 (8.7)
Restriction + Mixed	11 (29.7)	6 (6.0)	0 (–)	0 (–)	3 (4.5)	8 (2.7)
Obstruction	26 (70.3)	12 (12.0)	9 (25.7)	0 (–)	8 (11.9)	18 (6.4)
**IOS:** ***N*** **(%)**						
Abnormal IOS	18 (48.7)	104 (100)	13 (34.21)	0 (–)	22 (31.9)	82 (26.5)
Large airways	3 (8.1)	28 (26.9)	3 (7.9)	0 (–)	6 (8.7)	22 (7.1)
Small + small & large	15 (40.5)	76 (73.1)	10 (26.3)	0 (–)	16 (23.2)	60 (19.4)
**Exposure (ppb)/duration (years): Mean (Std) or** ***N*** **(%)**						
P95 Diacetyl	50.9 (76.2)	46.2 (56.1)	33.3 (39.5)	35.3 (37.4)	58.7 (80.9)	33.7 (29.8)
P95 2,3–Pentanedione	46.2 (91.6)	34.9 (64.3)	27.4 (49.3)	23.7 (33.9)	62.4 (102.7)	19.9 (12)
P95 Sum_DA+PD_	93.4 (165.1)	78.8 (116.6)	53.9 (54.9)	56.7 (65.2)	118.5 (179.5)	50.8 (32.3)
CE Diacetyl	50.5 (66.7)	65.5 (77.8)	43.4 (51.1)	53.1 (74.0)	70.7 (97.7)	56.1 (91.1)
CE 2,3–Pentanedione	36.7 (57.6)	45 (58.8)	35.5 (46.6)	34.5 (49.0)	58.9 (84.4)	33.4 (40.9)
CE Sum_DA+PD_	86.7 (125.4)	110.9 (135.6)	78.9 (95.9)	86.3 (117.6)	130.3 (183.1)	86.8 (110)
Avg. Diacetyl	13.0 (12.4)	15.5 (13.2)	8.78 (7.1)	12.7 (12.1)	13.4 (11.9)	13 (12.2)
Avg. 2,3–Pentanedione	9.2 (9.3)	9.9 (8.1)	7.7 (9.8)	7.9 (6.6)	10.9 (10)	7.9 (6.6)
Avg. Sum_DA+PD_	21.7 (21.0)	25.4 (20.9)	16.3 (15.5)	20.4 (18.4)	24.3 (20.9)	20.7 (18.3)
Total tenure (yrs.)	4.3 (3.7)	5.2 (5.4)	6 (5.3)	5.9 (6.1)	6.3 (5.8)	5.7 (6.1)
Flavoring tenure (yrs.)	0.9 (2.2)	0.9 (2.6)	0.95 (3)	0.4 (1.3)	2.8 (3.4)	0 (–)
Flavoring job	11 (29.7)	22 (21.2)	8 (21.1)	36 (16.0)	71 (100)	0 (–)
**Demographics:** ***N*** **(%) or Mean (Std)**						
Ever smoker	21 (56.8)	37 (35.6)	22 (57.9)	95 (42.2)	28 (39.4)	136 (43.5)
Age	37.7 (10.9)	37 (10.5)	38.8 (10.7)	36.6 (11.7)	36.3 (12.2)	37.2 (11.3)
Body Mass Index (BMI)	28.2 (7.4)	31.2 (6.5)	29.3 (6.5)	26.5 (4.8)	28.2 (6.1)	28 (5.9)
BMI ≥ 30	12 (32.4)	60 (57.7)	15 (39.5)	48 (21.3)	25 (35.2)	100 (32.1)
Race (White)	25 (67.6)	46 (44.2)	26 (68.4)	142 (63.1)	42 (59.2)	184 (58.8)
Gender (Male)	26 (70.3)	53 (51)	16 (42.1)	145 (64.4)	42 (59.2)	183 (58.5)

CE, cumulative exposure; Avg., average exposure; Severe SOB was assessed by a question on SOB occurring when walking with others of the same age.

**Figure 2:**
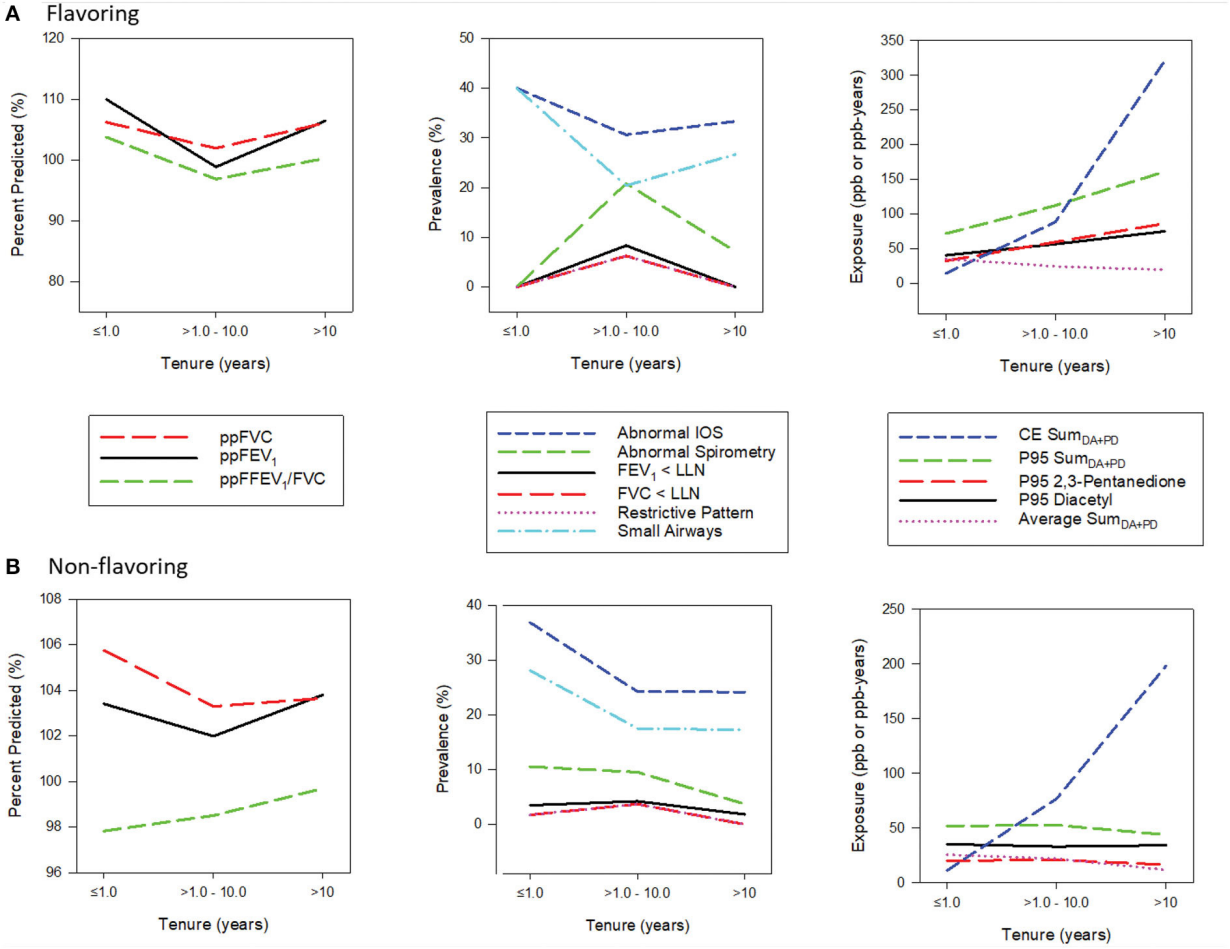
(A,B) Panel plots of exposure and health characteristics by tenure stratified by flavoring.

Figure 2 displays spirometric parameters, selected exposure metrics, and the prevalence of abnormal spirometry and IOS by categories of tenure and flavoring status; tenure categories were selected as ≤ 1 year representing short-tenure workers (17.5%), >1 to 10 years representing medium-tenure (63.5%) and >10 years representing long-term workers (19%). In the flavoring group, mean ppFEV_1_, ppFVC, and ppFEV_1_/FVC values decreased from the low to medium tenure category but increased in the high tenure category. Likewise, the prevalence of abnormal spirometry increased from the low to medium tenure category but decreased in the high tenure category. All exposure metrics, except for the average, show increasing trends with tenure. Similar patterns were observed in the non-flavoring group, albeit less pronounced, with all exposure metrics except cumulative exposure remaining flat across tenure. The prevalence of abnormal lOS decreased from low to medium tenure but increased or remained flat in the high category in both flavoring and non-flavoring groups.

### Exposure-response models for worklife exposure metric

#### P95 exposure

In exposure-response models for spirometry outcomes adjusted for covariates including tenure, the worklife P95 exposure metric for diacetyl, 2,3-pentanedione, and Sum_DA+PD_ was consistently associated with lower spirometric parameters; 2,3-pentanedione and Sum_DA+PD_ were significantly associated with lower ppFEV_1_ (Table 2). The parameter estimate for ppFEV_1_ with 2,3-pentanedione indicates a 0.30 percentage point lower ppFEV_1_ for every 10 ppb increase in P95 exposure. Elevated odds ratios were observed for any abnormal spirometry, FEV_1_ <LLN, FVC<LLN and restrictive pattern with P95 exposure for diacetyl, 2,3-pentanedione, and Sum_DA+PD_ in logistic models with covariates (Table 2). The OR (1.19) for the association of restrictive pattern with P95 diacetyl exposure is interpreted as a 19% increase in the odds of having a restrictive pattern for every 10 ppb increase in P95 exposure. Obstruction was not associated with any exposure metrics. Significantly elevated odds ratios were observed for abnormality in the small airways with P95 exposure, for diacetyl, 2,3-pentanedione, and Sum_DA+PD_ in logistic models with covariates; overall abnormal IOS followed a similar pattern, albeit with smaller odds ratios (Table 2). The increase in odds for these associations ranged from 3 to 8% for having IOS or small airway abnormalities for every 10 ppb increase in exposure. Large airway abnormality was not associated with any exposures.

**Table 2:** Associations of lung function with worklife P95 exposure metric for diacetyl, 2,3-pentanedione, and Sum_DA+PD_.

**Health outcome**	**Diacetyl**	**2,3-Pentanedione**	**Sum_DA+PD_**
	**slope (95% CI)**	**slope (95% CI)**	**slope (95% CI)**
ppFEV_1_	−0.24 (−0.55, 0.07)	**−0.30** **(−0.58**, **−0.03)**	**−0.16** **(−0.32**, **−0.01)**
ppFVC	−0.21 (−0.50, 0.08)	*−0.25* *(−0.51, 0.01)*	*−0.14* *(−0.28, 0.01)*
ppFEV_1_/FVC	−0.05 (−0.25, 0.16)	−0.08 (−0.26, 0.10)	−0.04 (−0.14, 0.06)
	**OR (95% CI)**	**OR (95% CI)**	**OR (95% CI)**
FEV_1_ < LLN	**1.11** **(1.01, 1.21)**	**1.11** **(1.03, 1.19)**	**1.06** **(1.02, 1.10)**
^a^FVC < LLN	**1.19** **(1.09, 1.31)**	**1.12** **(1.05, 1.20)**	**1.06** **(1.03, 1.12)**
FEV_1_/FVC < LLN	1.05 (0.96, 1.14)	**1.06** **(0.99, 1.12)**	**1.04** **(0.99, 1.07)**
Abnormal spirometry	**1.08** **(1.01, 1.16)**	**1.08** **(1.02, 1.14)**	**1.04** **(1.01, 1.08)**
^a^Spirometry obstruction	1.00 (0.86, 1.11)	1.04 (0.94, 1.12)	1.02 (0.95, 1.06)
^a^Spirometry restriction + Mixed	**1.19** **(1.09, 1.32)**	**1.13** **(1.05, 1.21)**	**1.08** **(1.03, 1.13)**
^b^Abnormal IOS	*1.06* *(1.00, 1.12)*	*1.05* *(0.99, 1.10)*	*1.03* *(1.00, 1.06)*
^a, b^IOS Large airways	1.00 (0.88, 1.10)	0.99 (0.80, 1.08)	1.00 (0.92, 1.05)
^a, b^IOS small + small and Large airways	**1.08** **(1.02, 1.15)**	**1.06** **(1.01, 1.12)**	**1.04** **(1.01, 1.07)**

Covariates, age, BMI, height, tenure, sex, smoke, race, and allergic status; Estimates of slope and their 95% CI are expressed as percentage points per 10 ppb; Estimates of OR and their 95% CI are expressed as odds per 10 ppb; Bold, p < 0.05; Italics, 0.05 < p < 0.10; Models modified to address quasi or complete separation. All logistic and polytomous models used binary race, whites vs. non-whites;

a sex excluded;

b height included.

#### Average and cumulative exposures

Average and cumulative exposure to diacetyl, 2,3-pentanedione, and Sum_DA+PD_ were consistently associated with lower ppFVC but not with ppFEV_1_ and ppFEV_1_/FVC (Supplementary Table 1). Most notably, FVC<LLN and restrictive spirometric patterns were significant across all exposure metrics. FEV_1_ < LLN was significant for 2,3-pentanedione for average and cumulative exposure. Measures of IOS abnormalities, including small and large airway abnormalities, had odds ratios that were much smaller in magnitude with large confidence intervals.

#### Model covariates

Various covariates were significant for different spirometry and IOS outcomes (Supplementary Table 2) and are described in the Supplementary materials. Model fit and precision metrics are reported in Supplementary Table 3. Duration of exposure (tenure) was positively associated with all continuous spirometry outcomes, indicating significantly higher spirometric values with increasing tenure. Tenure was thus included as a covariate in all the models. There was no interaction between tenure and the exposure metrics. However, a significant interaction was observed between flavoring status and the exposure metrics, thus stratified analyses were conducted.

### Exposure-response models stratified by flavoring status

In models stratified by flavoring status, the highest-P95 and cumulative exposure metric for diacetyl, 2,3-pentanedione, and Sum_DA+PD_ were associated with lower ppFEV_1_, ppFVC, and ppFEV_1_/FVC in the flavoring group and were significant for the association between highest-P95 and ppFEV_1_ and ppFVC (Supplementary Table 4). Diacetyl exposures had the largest effect estimates, which were larger than those for the overall model. None of the exposure metrics were associated with ppFEV_1_ or ppFVC in the non-flavoring group (Supplementary Table 5). Diacetyl, 2,3-pentanedione, and Sum_DA+PD_ were associated with elevated ORs for FEV_1_ <LLN, FVC<LLN, and restrictive patterns in the flavoring group, at *p* < 0.05 or 0.05 < *p* < 0.1 (Supplementary Table 4). This is likely due to the small sample size in the flavoring group (*n* = 71), with only 11 cases of abnormal spirometry and three with a restrictive pattern. In the non-flavoring group, FVC<LLN and restrictive pattern were associated with elevated ORs for highest-P95 and cumulative exposures at *p* < 0.05 or 0.05 < *p* < 0.1 (Supplementary Table 5); there were 26 cases of abnormal spirometry and eight with restrictive pattern in the non-flavoring group of *n* = 313 workers. For IOS in the flavoring group, significant ORs were observed for small airway abnormality with the highest-P95 for diacetyl, 2,3-pentanedione, and Sum_DA+PD_ (Supplementary Table 4); no associations were observed in the non-flavoring group (Supplementary Table 5).

### Exposure-response models for current exposure metrics

None of the metrics of current exposures based on full-shift, short-duration task-based, or instantaneous activities were associated with any IOS or spirometry outcomes (data not shown).

## Discussion

### Association of α-diketone exposures with lung function decrements and abnormalities

Decrements in ppFEV_1_ and ppFVC were consistently associated with increasing highest-P95 exposure to diacetyl, 2,3-pentanedione, and Sum_DA+PD_ overall or in flavoring. Average and cumulative exposure metrics were also consistently inversely associated with lung function parameters, albeit non-significantly. Elevated ORs for FEV_1_ <LLN, FVC<LLN, abnormal spirometry, and restrictive patterns were observed overall, in flavoring, and non-flavoring for highest-P95, cumulative, and average exposure to all the α-diketones. Additionally, the combined categories of small and peripheral plus small and large airway abnormalities on IOS had elevated ORs for highest-P95 exposure to the α-diketones. These associations were observed with worklife exposure metrics but not with current exposures. Highest-P95, a surrogate of peak exposure, seemed to be a more sensitive metric, but cumulative and average exposure was also significant. The association of α-diketone exposures with lung function decrements and abnormalities in a workforce with relatively few lung function abnormalities (7) indicates a potential risk for future occupational lung disease at these facilities with prolonged exposures. Previous studies in flavoring workers have observed increasing symptoms and lung function abnormalities prior to the development of OB (25). Symptoms of chronic respiratory impairment and respiratory abnormalities, one case of OB identified in this workforce (9), and the observed associations with α-diketone exposures in this study may be indicative of early disease markers.

In the past, OB was described as fixed airway obstruction, with spirometry measures focused on obstruction. However, recent studies using spirometry have reported obstruction, restrictive pattern, and mixed obstruction and restriction in popcorn and flavoring manufacturing workers (11, 15, 19). In one flavoring manufacturing facility, abnormal spirometry was mostly restrictive but also included obstructive and mixed patterns (20). Obstruction, restrictive, and mixed patterns on spirometry were also observed in the present workforce, but restrictive pattern was significantly associated with α-diketone exposure metrics. The restrictive pattern is consistent with the findings of small airway abnormalities on IOS (15, 38–40), which was also significantly associated with the highest-P95 exposure to α-diketones.

Exposure–response relationships observed for continuous spirometric outcomes suggest that the effect of exposure on lung function occurs in the entire workforce and is not limited to just the subpopulation with sufficient loss of function to be classified as abnormal. However, continuous outcome variables may be affected by variability in spirometric values, resulting in wider confidence intervals, or nonlinear relationships between lung function values and exposure metrics.

### Effect of various exposure metrics for diacetyl, 2,3-pentanedione, and Sum_**DA+PD**_

The effect estimates for the associations between various health outcomes and exposure to diacetyl and 2,3-pentanedione when both estimates were significant were similar, but diacetyl had slightly larger effect estimates than 2,3-pentanedione for the highest-P95 metric. The converse was true for average and cumulative exposure metrics. There was a high correlation between diacetyl and 2,3-pentanedione for highest-P95 (ρ_s_ = 0.89), cumulative (ρ_s_ = 0.97), and average (ρ_s_ = 0.94) exposure metrics. Therefore, it is not possible to evaluate the independent effect of each α-diketone as the effect estimate reflects the effect of the combination of the two. Because of the high correlation between diacetyl and 2,3-pentanedione, neither remains significant when included in the same model and their interaction (to determine additive or multiplicative effect) could not be evaluated (41). The effect estimate for the sum of the two α-diketones is essentially half of the estimate for diacetyl or 2,3-pentanedione because the range of the exposure estimate has been doubled. The effect estimate reflects the combined effects of the α-diketones; the individual α-diketones underestimate the exposure and therefore overstate the risk, whereas considering the sum of the α-diketones reduces this underestimation of exposures. If the effect of diacetyl is similar to that of 2,3-pentanedione (11, 22), then the effect estimate of the sum of the α-diketones may be more representative of the mixed exposure effect than the individual α-diketones.

Summarizing time-varying historical exposure profiles into summary exposure metrics for use in epidemiologic studies involves assumptions about the relationship between exposure and disease and the time patterns of the effects of exposure (42, 43). Cumulative exposure is the most common exposure index used in epidemiologic studies of chronic effects; however, metrics of peak exposure may be relevant when the association between exposure, dose, and impairment is nonlinear (43, 44). In this study, high to moderate correlations were observed for the highest-P95 and the average exposure (ρ_s_ = 0.86), between average and cumulative (ρ_s_ = 0.45), and highest-P95 and cumulative (ρ_s_ = 0.50) exposure for the sum of the two α-diketones. Although only the highest-P95 was significantly associated with continuous spirometric parameters, all three, i.e., highest-P95, average, and cumulative exposure metrics, were associated with the categorical spirometric outcomes, perhaps reflecting more complex exposure–response relationships or measurement errors in exposure or outcome variables. Differences in the exposure–response relationship have been observed even when the summary exposure metrics are highly correlated (*r* = 0.68 to 0.88) (45).

### Effect of flavoring status

Average values for FEV_1_ and FVC were similar between flavoring and non-flavoring groups. However, a significant interaction was observed between flavoring status and exposure metrics. Highest-P95 metric for α-diketone exposures was strongly associated with decrements in the continuous spirometric parameters overall and in flavoring, but not in non-flavoring; the effect estimate was larger in flavoring than overall. All exposure metrics were higher in flavoring compared to non-flavoring, although this is accounted for in the exposure metric used in regression models. There are likely other factors contributing to the difference in effect between the groups, such as other co-exposures in the flavoring group or differences in exposure time needed to experience lung function decrements given the lower exposures in non-flavoring. Abnormal spirometry, including obstruction and restrictive pattern, occurred in both flavoring and non-flavoring groups. Although some of the associations in the flavoring and non-flavoring groups were not significant, these associations are affected by sample size and the number of cases with abnormality, with only three cases of restrictive pattern in flavoring and eight cases in non-flavoring. These findings indicate that α-diketone-related spirometric abnormalities occur in both flavoring and non-flavoring workers.

The effect estimates reported in the NIOSH criteria document for α-diketones are for FEV_1_, the ratio of FEV_1_/FVC, and obstruction-associated average and cumulative exposure to diacetyl in flavoring (11). In this study, the associations of FEV_1_ or the ratio of FEV_1_/FVC with average or cumulative diacetyl exposure were not significant. Additionally, obstruction was not significantly associated with any of the exposure metrics. Thus, the present results cannot be directly compared to those reported in the NIOSH criteria document. It is noteworthy that the effect estimates in this study are in units of 10 ppb^−1^, while those reported in the criteria document are in ppm^−1^.

### Healthy worker effect

The plots of lung function parameters presented in Figure 2 show worsening spirometry going from a low tenure of <1 year to a medium tenure of 1–10 years, followed by improved spirometry in the highest tenure of >10 years. All exposure metrics, except for the average metric, increase with increasing tenure or remained flat (for non-flavoring). Additionally, regression models with tenure as the main effect (without exposures) showed tenure was significantly associated with better spirometry for some outcomes, such as FEV_1_ and the ratio of FEV_1_/FVC. These findings indicate a potential for a healthy worker survivor effect, and tenure was thus included as a covariate in all the models to account for this positive effect on spirometry. In a study of flavoring manufacturing workers with a higher prevalence of respiratory symptoms, a positive association was also observed between the duration of work in the diacetyl plant and ppFEV_1_, which was attributed to various causes, including the healthy worker effect (17). This phenomenon is not uncommon in occupational studies where the effect estimate is attenuated in the higher exposure category because of various potential causes, including the healthy worker survivor effect, fewer susceptible workers in the population at high exposure levels, measurement error or exposure misclassification, and the influence of other risk factors that are correlated with exposure (46).

### Limitations

With any approach, there are trade-offs and limitations of the selected study design or strategy. Some limitations of this study included a lack of historical exposure information; historical job exposures were assumed to be equal to current exposures; historical task information was not gathered; co-exposures to other gases, dust, and vapors in coffee production might also be important but were not collected; a small number of cases of spirometric abnormalities; and other general limitations of cross-sectional study design. Smaller sample sizes may have contributed to the lack of significance found in some of these analyses. Additionally, although the health assessment was extensive, there were several limitations, including the inability to assess longitudinal change in lung function because of the cross-sectional study design, the potential for healthy worker survivor effect because of enrolling current workers only, the potential for bias if differential participation by health status occurred because participation was not 100%, and potential for underestimation of exposure and respiratory health burden in the industry as the HHE requests were often made by management at facilities without known health problems. Some of these challenges could not be avoided within the HHE Program context.

## Conclusion

Lung function decrements and abnormalities were consistently associated with various metrics of exposure to diacetyl, 2,3-pentanedione, and their sum in a workforce of coffee production workers with relatively few workers with lung function changes large enough to be classified as abnormal and a likely presence of healthy worker survivor effect. Although obstruction, restrictive, and mixed spirometric patterns were present, only restrictive plus mixed pattern was significantly associated with α-diketones exposures, consistent with the association for small airway abnormality. The effects of exposure likely occur in the entire population and not just among workers with lung function abnormalities or just in flavoring. Although the highest-P95 summary metric appeared to be more sensitive, average and cumulative exposure metrics are also relevant. An aggregate exposure metric ought to be considered when multiple α-diketones are present. Associations between lung function abnormalities and exposure to α-diketones suggest it may be prudent to consider exposure controls in both flavoring and non-flavoring settings.

## Data availability statement

The datasets presented in this article are not readily available because due to restrictions imposed under the US privacy act and the limitations of what participants consented to, the data underlying the analyses presented, beyond what is provided in the paper, are confidential and not available to researchers outside the National Institute for Occupational Safety and Health (NIOSH). For more information about NIOSH's policy regarding sensitive data, see https://www.cdc.gov/niosh/ocas/datahandle.html. Requests to access the datasets should be directed to https://www.cdc.gov/niosh/ocas/datahandle.html.

## Ethics statement

The studies involving human participants were reviewed and approved by the NIOSH Institutional Review Board reviewed and approved this study (NIOSH Protocol 17-RHD-06XP). All participants provided their written informed consent to participate during data collection for the HHE.

## Author contributions

MV, JC-G, and KC contributed to the conception and design of the study. XL organized the database. MV, JC-G, KC, EF-L, RH, and RB contributed to the interpretation of IOS and spirometry results. MV, BB, RL, and MS contributed to the estimation of exposure. MV, XL, and CG performed the statistical analyses. MV wrote the first draft of the manuscript. All authors contributed to the article and approved the submitted version.

## Funding

This study was supported by the National Institute for Occupational Safety and Health (NIOSH) Division Intramural Funds.

## Conflict of interest

The authors declare that this study was conducted in the absence of any commercial or financial relationships that could be construed as a potential conflict of interest.

## Publisher's note

All claims expressed in this article are solely those of the authors and do not necessarily represent those of their affiliated organizations, or those of the publisher, the editors and the reviewers. Any product that may be evaluated in this article, or claim that may be made by its manufacturer, is not guaranteed or endorsed by the publisher.

## Author disclaimer

The findings and conclusions in this report are those of the author(s) and do not necessarily represent the official position of the National Institute for Occupational Safety and Health, Centers for Disease Control and Prevention.
