# Supplementary material for: Decrements in lung function and respiratory abnormalities associated with exposure to diacetyl and 2,3-pentanedione in coffee production workers
Source: Front Public Health. 2022 Aug 12;10:966374. doi: 10.3389/fpubh.2022.966374 (PMC9412051; doi:10.3389/fpubh.2022.966374)
Supplement: Supplementary file 1 [file Data_Sheet_1.PDF]

Supplementary Material

Decrements in lung function and respiratory abnormalities associated with exposure to diacetyl and 2,3-pentanedione in coffee production workers

M. Abbas Virji[1]*, Ethan D. Fechter-Leggett[1], Caroline P. Groth[2], Xiaoming Liang[1], Brie H. Blackley[1], Marcia L. Stanton[1], Ryan F. LeBouf[1], R. Reid Harvey[1], Rachel L. Bailey[1], Kristin J. Cummings[1], Jean M. Cox-Ganser[1]

[1]Respiratory Health Division, National Institute for Occupational Safety and Health, Centers for Disease Control and Prevention, Morgantown, WV USA

[2]Department of Epidemiology and Biostatistics, School of Public Health, West Virginia University, Morgantown, WV, United States

1.1 Supplementary Methods

Impulse Oscillometry

Impulse oscillometry (IOS) was performed using CareFusion IOS system (CareFusion, Hochberg, Germany) according to the manufacturer's instructions. IOS can augment spirometry as a more sensitive metric of small airways dysfunction (1) and may serve as an early indicator of respiratory dysfunction by identifying pulmonary abnormalities in workers with normal spirometry (2, 3). IOS measures the mechanical properties of the respiratory system including upper and intrathoracic airways, lung tissue, and chest wall (4, 5). Respiratory impedance, the sum of forces that oppose the impulse generated (force needed to move gas into and out of the airways), consists of resistance (R_{Hz}), which is the resistance of the conducting airways to the flow of the gas, and reactance (X_{Hz}), which includes elasticity (elastic recoil) of the lungs measured in capacitance and inertia of the gas (1, 4). IOS parameters include: 1) resistance at oscillation frequency of 5 Hz (total resistance – small and large airways) and 20 Hz (proximal resistance – large airways) (R_5, R_{20}); 2) frequency dependence of resistance obtained as the difference between R_5 and R_{20} ($R_{5–20}$); 3) reactance at 5 Hz (distal capacitance – peripheral) (X_5); 4) resonant frequency (f_{res}); and 5) reactance area (AX) calculated as the area under the reactance curve from 5 Hz to f_{res} (6). Percent difference R_5-R_{20} (DR_{5-20}) is calculated as ((R_5-R_{20})/R_{20})*100%; ppR_5 is percent predicted R_5. Small airways and peripheral abnormality was defined as ($DR_{5–20} \geq 30\%$) or ($ppR_5 \geq 140\%$, [$X_{5\ predicted}$ - $X_{5\ measured}$] $\geq$ 0.15 kPa/(L/s) and $DR_{5–20} \geq 30\%$), or ($ppR_5 < 140\%$ and [$X_{5\ predicted}$ - $X_{5\ measured}$] $\geq$ 0.15 kPa/(L/s)); large and central airways abnormality was defined as ($ppR_5 \geq 140\%$, [$X_{5\ predicted}$ - $X_{5\ measured}$] < 0.15 kPa/(L/s) and $DR_{5–20} < 30\%$); small and large airways abnormality was defined as ($ppR_5 \geq 140\%$, [$X_{5\ predicted}$ - $X_{5\ measured}$] $\geq$ 0.15 kPa/(L/s) and $DR_{5–20} < 30\%$); and any IOS abnormality was defined as $ppR_5 \geq$ 140% or [$X_{5\ predicted}$ - $X_{5\ measured}$] $\geq$ 0.15 kPa/(L/s) (7, 8). For data analysis, small airways and peripheral abnormality were combined with small and large airways abnormality to emphasize any abnormality involving small airways.

Exposure Summaries for a Job/Task Exposure Matrix (JEM/TEM)

Personal full-shift, and short-duration and instantaneous task-based sampling was conducted for alpha-diketones at all 17 facilities as described in detail elsewhere (9). These measurements were used to generate a job- and task-exposure matrix (JEM/TEM), and to assign exposure estimates to participants based on the work histories reported in the questionnaire, as outlined in Figure 1 (in the main text). The sampling and analysis approach enabled: 1) quantification of diacetyl and 2,3-pentanedione to evaluate the individual or combined alpha-diketones effect on respiratory health, and 2) development of multiple exposure metrics to test different hypotheses of the effect of peak, average or cumulative exposure on health.

Full-shift, task-based and instantaneous exposure measurements (depicted in the first column of Figure 1 - in the main text) were quantified in parts per billion (ppb) and summarized overall, as well as stratified by facility, facility size category (>10,000 pounds = large, 10,000–1,000 pounds = medium and <1000 pounds of coffee processed per day = small) and flavoring status (flavored vs. did not flavor coffee) using a Bayesian approach (9). This approach fits a repeated measures analysis of variance (ANOVA) model which accounts for censored data and

repeated measurements collected on workers when at least five workers are present and at least two workers have repeated measurements; otherwise, a fixed effects model is fit. The median posterior estimate of the mean, standard deviation and P95 of the log-transformed exposures were obtained from these models, and were used to calculate the minimum variance unbiased estimator (MVUE) of the arithmetic means (AM); the MVUE is the preferred estimator of the AM when the data come from a lognormal distribution (10).

A JEM was constructed that included the AM and P95 for diacetyl, 2,3-pentanedione, and the sum of diacetyl and 2,3-pentanedione for all jobs, overall and stratified by facility, facility size category and flavoring status. Likewise, the TEM included the AM and P95 for diacetyl, 2,3-pentanedione, and the sum of diacetyl and 2,3-pentanedione for all short-duration tasks, overall and stratified by facility, facility size category and flavoring status. The TEM also included the P95 for diacetyl, 2,3-pentanedione, and the sum of diacetyl and 2,3-pentanedione for the instantaneous activities associated with each task, overall and stratified by facility, facility size category and flavoring status as depicted in the second column of Figure 1 (in the main text). The JEM/TEM included only estimates based on current exposures as historical exposure data have not previously been collected at coffee production facilities. Based on knowledge of the industry and discussions with facility owners, the assumption was made that historical exposures at these facilities were similar to current exposures because there were no systematic changes in the industry that may impact exposures. Flavoring ingredients likely changed from diacetyl to 2,3-pentanedione which would change the proportion of each chemical, but not the total α-diketones.

Exposure Assignment and Summary Metrics

Information on jobs performed in the coffee or flavoring industry and the tenure at each job was gathered in the work history part of the questionnaire. Additionally, the exposure module elicited information on the frequency and duration of tasks performed in current job, however, the duration and frequency of tasks performed in previous jobs was not gathered because of the high potential for error or bias in recalling such detailed information about tasks in past jobs (11). The AM and P95 from the JEM were then assigned to all the jobs reported by each participant in their work history, starting with the most specific facility level exposure estimates. If exposure estimates were unavailable at the facility level, estimates from facility size category were assigned, followed by estimates from flavoring status, and ending with the most general overall (unstratified) estimates. The AM and P95 of the most recent job were classified as average and highest "current exposure" metrics in ppb (depicted in the third column, first row of Figure 1 - in the main text). The profile of AM and P95 from multiple jobs held by workers were summarized to obtain worklife highest, average and cumulative exposure summary metrics for each worker. The highest P95 from all jobs held by a worker was selected as the worklife highest exposure summary metric in ppb. The AMs associated with each job in the work history were then multiplied by the duration (in years) that the job was performed for and summed across all jobs held to obtain worklife cumulative exposure summary metric in ppb-years; the cumulative exposure was divided by total work tenure to obtain worklife average exposure summary metric in ppb (depicted in the fourth column, first row of Figure 1 - in the main text). The AM is the toxicologically-relevant metric to calculate worklife average and cumulative exposure summary metrics (12).

The AM and P95 from the TEM were then assigned to all the tasks and activities reported by each participant in the exposure modules of the questionnaire, following the same order as described for assigning job exposures. The highest P95 of all the tasks or all the activities were selected as the highest short-duration or instantaneous peak “current exposure” metrics in ppb (depicted in the third column, third row of Figure 1 - in the main text). The duration (hours per day) and frequency (days per week) of tasks performed reported in the exposure modules was used to calculate a task weight as:

Task weight = [Duration (hours/day) x Frequency (days/week)]/40 (hours/week).

The AMs of the profile of short-duration tasks performed by a worker were averaged to create average unweighted short-duration current exposure metric in ppb; the task AMs were also multiplied by the task weights and summed to create average weighted short-duration current exposure metric in ppb (depicted in the third column, third row of Figure 1 - in the main text). Worklife exposure metrics could not be created for task-based exposures because historical task information was not collected.

The summary metrics created included: the highest instantaneous P95 exposure for current activities; the highest P95 and the average short-duration exposures for current tasks, weighted or unweighted by task duration and frequency; the highest (P95) and average full-shift exposure for current job; and highest (P95), average and cumulative exposures for all jobs as worklife metrics. These summary metrics were calculated for diacetyl, 2,3-pentanedione, the sum of the two as α-diketones. These wide range of metrics offer the opportunity to thoroughly examine the effects of current or worklife exposure to specific α-diketones and their sum, peak, average or cumulative exposure metrics on various respiratory health outcomes of interest to explore multiple hypotheses about the nature of exposure-response relationships.

1.2 Supplementary Results and Discussion

Effects of Co-variates on Lung Function Measures

Various covariates were significant for different spirometry and IOS outcomes, some of which are also accounted for in the reference equations. Significant covariates included BMI, tenure and sex for ppFEV$_1$, BMI and sex for ppFVC, and age, tenure, smoking status and race for ppFEV$_1$/FVC for diacetyl, 2,3-pentanedione and Sum$_{DA+PD}$(Supplementary Table S2). Tenure was significant for abnormal spirometry, only BMI was significant for FVC<LLN and restrictive pattern, and BMI, sex and smoking status were significant for FEV$_1$<LLN (Supplementary Table S2). Only BMI and height were significant covariates for abnormal IOS and small airways abnormality (Supplementary Table S2).

Where age, body mass index (BMI) and ever-smoking status were significant, they were inversely associated with the continuous spirometry outcomes and had elevated odds ratios for categorical spirometry or IOS outcomes. Tenure and height were positively associated with the continuous spirometry outcomes and had lowered odds ratios for categorical spirometry or IOS outcomes. Female sex and Hispanic or other race (compared to white race) were positively associated with the continuous spirometry outcomes, and female sex had lowered odds ratios for categorical spirometry outcomes; race was not included in most categorical models because of separation errors in the models. Allergic status was not significant in any models. Whereas

tenure was significant in some models, excluding it affected the exposure-response effect estimate and was thus retained in all models. In models where a covariate was excluded because of separation errors, keeping the variable in the model slightly attenuated the effects estimate for the exposure metric, but did not alter the level of significance. BMI, tenure, and smoking status are not included in spirometry reference equations and had a significant impact on the outcome variables; age, sex and race are accounted for in the reference equations but nonetheless were significant in some of the models. For IOS, BMI and height were significant in many of the models but are not accounted for in the reference equations. Reference equations are being developed that account for BMI and height (6).

Model fit and precision

Measures of model fit (AIC) and precision of the parameter estimate (β/SE) are summarized in Supplementary Table S3 for each model. AIC was similar across all models but was often the lowest for 2,3-pentanedione, followed by Sum_{DA+PD} and diacetyl; the precision of the parameter estimate followed the same pattern for the significant exposure variables (Supplementary Table S3). Model fit and precision metrics were best for 2,3-pentanedione, followed by Sum_{DA+PD} and diacetyl for most outcomes except FVC<LLN and restrictive pattern which has slightly better model fit and precision metrics for diacetyl (Supplementary Table S3). Model fit and precision metrics for IOS and small airway abnormalities were slightly better for diacetyl, followed by Sum_{DA+PD} and 2,3-pentanedione for the P95 exposure metric (Supplementary Table S3).

Supplementary Figure S1: Distributions and summary statistics of worklife exposure metrics for diacetyl, 2,3-pentanedione, Sum_{DA+PD} (ppb) and lung function parameters.

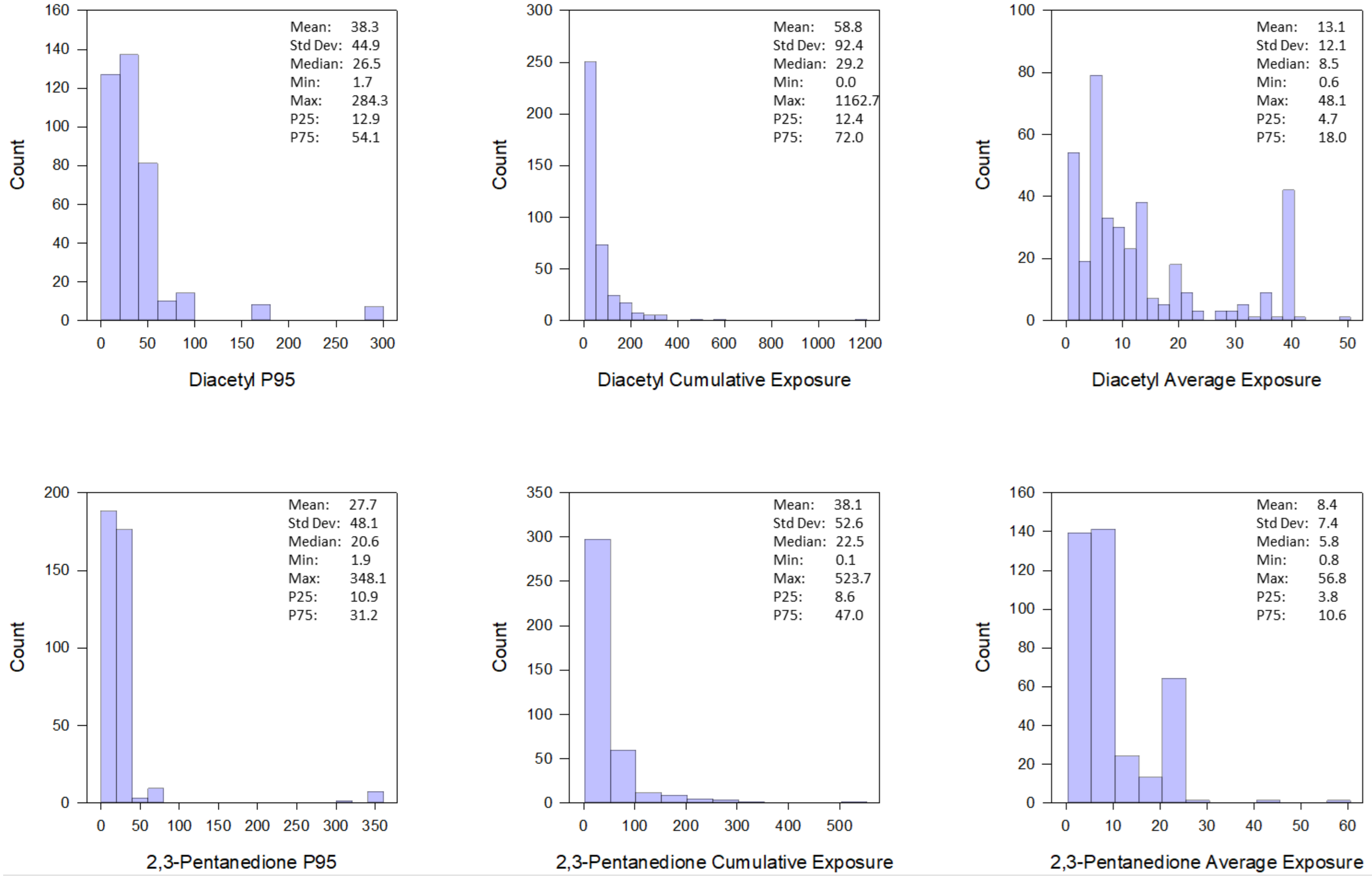

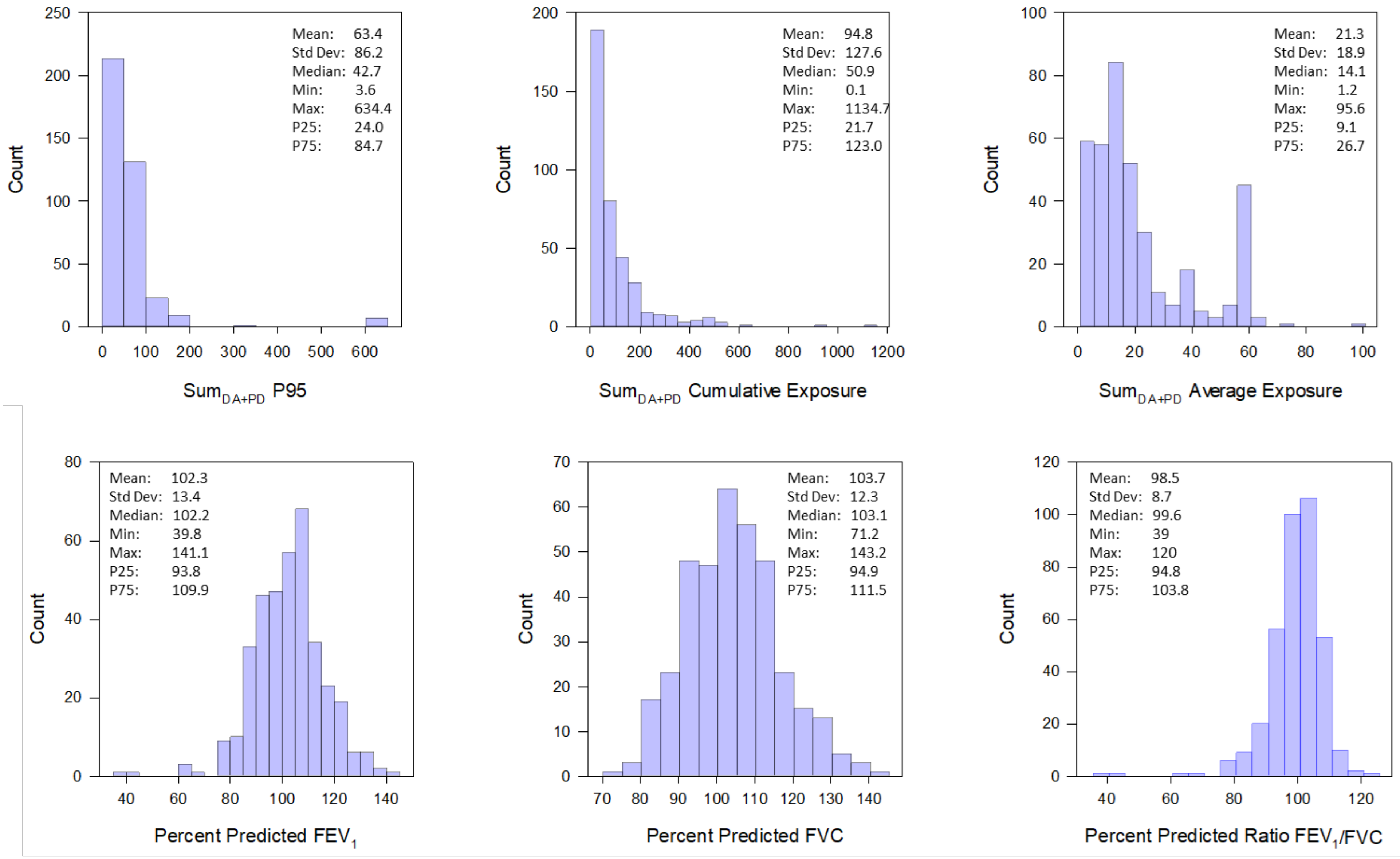
Count
Mean: 63.4
Std Dev: 86.2
Median: 42.7
Min: 3.6
Max: 634.4
P25: 24.0
P75: 84.7
Sum DA+PD P95
Count
Mean: 94.8
Std Dev: 127.6
Median: 50.9
Min: 0.1
Max: 1134.7
P25: 21.7
P75: 123.0
Sum DA+PD Cumulative Exposure
Count
Mean: 21.3
Std Dev: 18.9
Median: 14.1
Min: 1.2
Max: 95.6
P25: 9.1
P75: 26.7
Sum DA+PD Average Exposure
Count
Mean: 102.3
Std Dev: 13.4
Median: 102.2
Min: 39.8
Max: 141.1
P25: 93.8
P75: 109.9
Percent Predicted FEV1
Count
Mean: 103.7
Std Dev: 12.3
Median: 103.1
Min: 71.2
Max: 143.2
P25: 94.9
P75: 111.5
Percent Predicted FVC
Count
Mean: 98.5
Std Dev: 8.7
Median: 99.6
Min: 39
Max: 120
P25: 94.8
P75: 103.8
Percent Predicted Ratio FEV1/FVC

Supplementary Figure S2: A heatmap plot of correlation coefficients among all exposure metrics

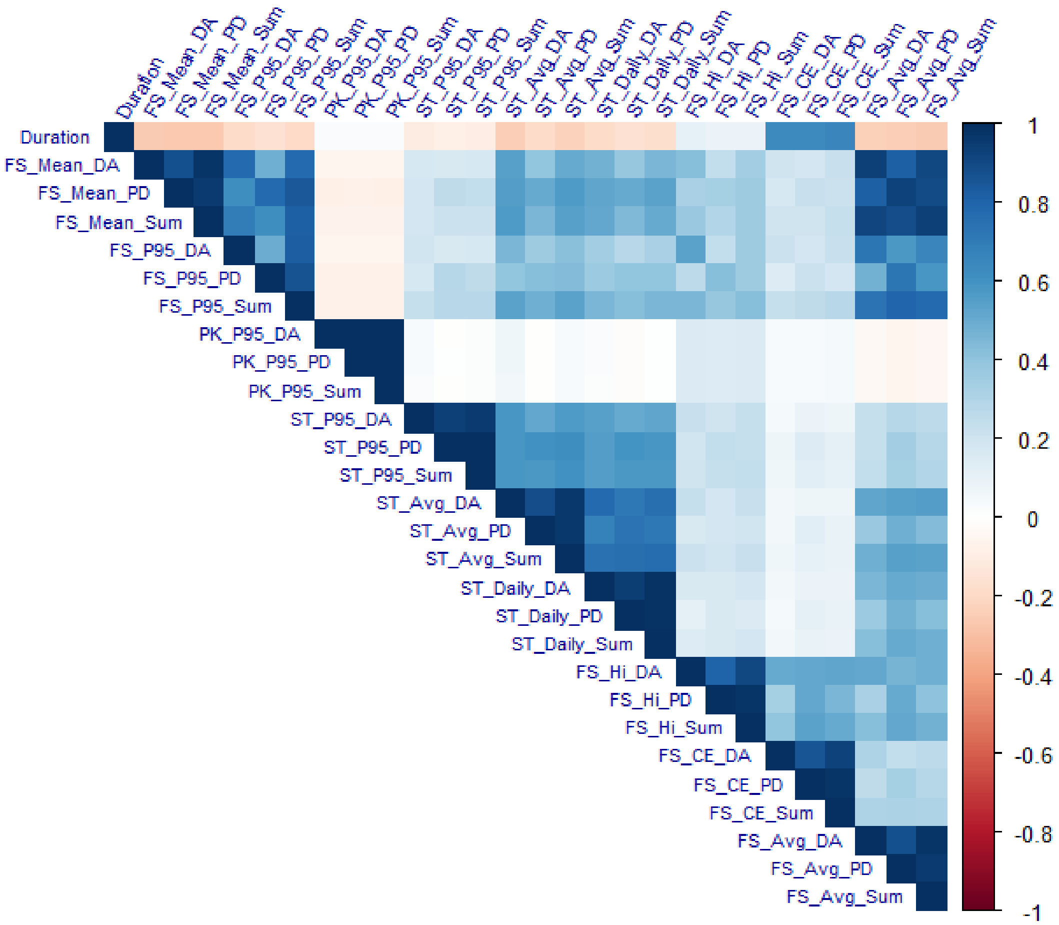

Supplementary Figure S3: Scatter plots displaying correlations among summary metrics for diacetyl and 2,3-pentanedione and their sum

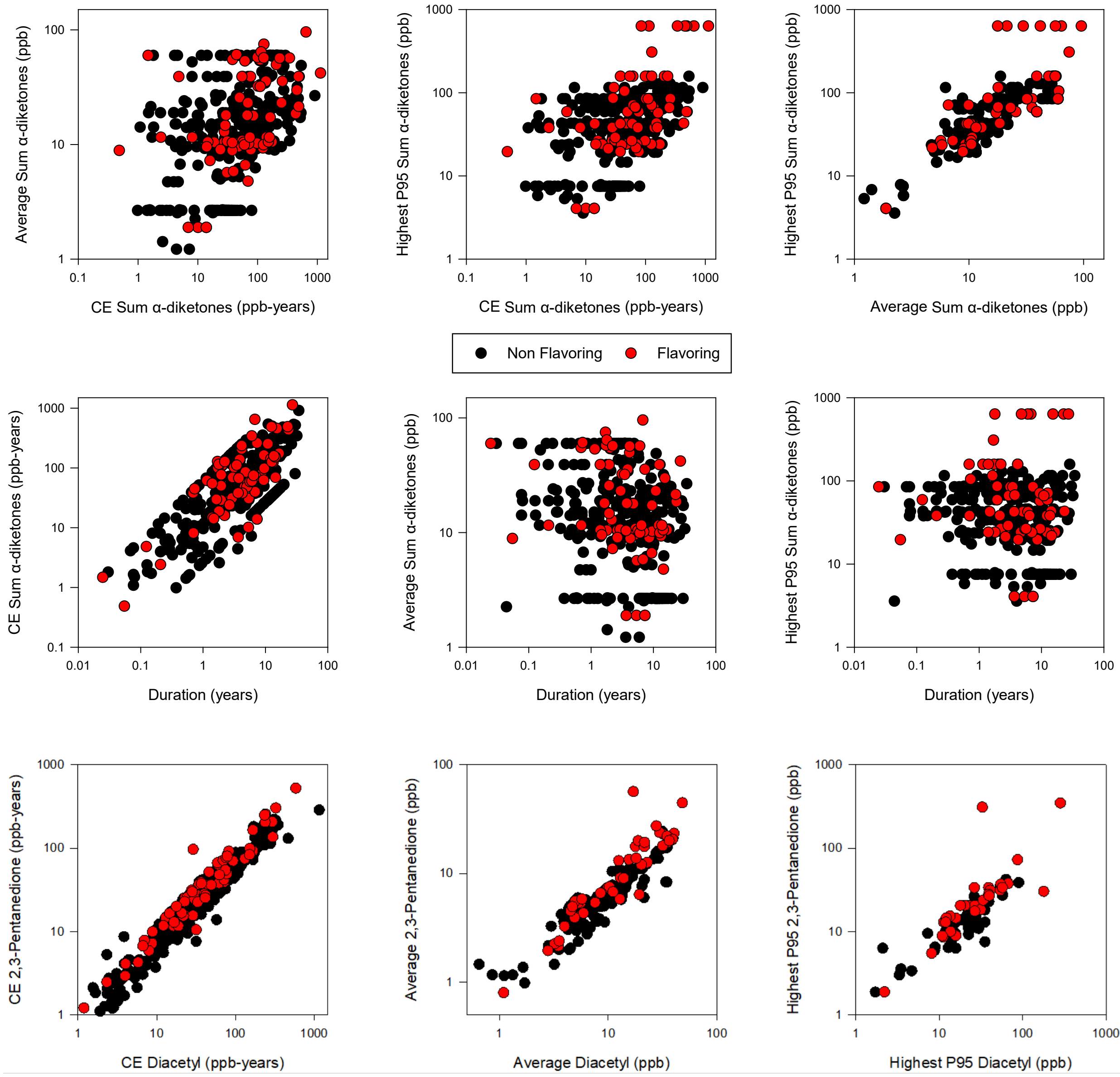

Supplementary Tables

Supplementary Table S1: Associations of lung function with worklife average and cumulative exposure metrics to diacetyl, 2,3-pentanedione, and Sum$_{DA+PD}$

Health Outcome	Diacetyl		2,3-Pentanedione		Sum$_{DA+PD}$	
	Average Slope (95% CI)	CE Slope (95% CI)	Average Slope (95% CI)	CE Slope (95% CI)	Average Slope (95% CI)	CE Slope (95% CI)
ppFEV$_1$	0.14 (-1.21, 1.49)	0.02 (-0.23, 0.27)	-0.08 (-2.16, 2.01)	-0.15 (-0.50, 0.21)	0.01 (-0.84, 0.87)	-0.04 (-0.19, 0.11)
ppFVC	-0.51 (-1.76, 0.73)	-0.11 (-0.34, 0.12)	-1. 03 (-2.95, 0.89)	*-0.27 (-0.60, 0.05)*	-0.37 (-1.16, 0.42)	-0.10 (-0.24, 0.04)
ppFEV$_1$/FVC	0.64 (-0.23, 1.51)	0.12 (-0.04, 0.28)	0.86 (-0.48, 2.20)	0.10 (-0.13, 0.33)	0.36 (-0.19, 0.91)	0.05 (-0.05, 0.15)
	Average OR (95% CI)	CE OR (95% CI)	Average OR (95% CI)	CE OR (95% CI)	Average OR (95% CI)	CE OR (95% CI)
FEV$_1$ < LLN	1.21 (0.72, 1.98)	1.04 (0.95, 1.13)	**1.90 (1.01, 3.37)**	**1.11 (0.99, 1.22)[c]**	1.24 (0.91, 1.68)	1.04 (0.99, 1.09)
[a]FVC < LLN	**1.92 (1.13, 3.41)**	**1.19 (1.07, 1.36)**	**2.32 (1.26, 4.43)**	**1.26 (1.10, 1.53)**	**1.50 (1.09, 2.10)**	**1.11 (1.04, 1.21)**
FEV$_1$/FVC < LLN	0.79 (0.48, 1.21)	1.02 (0.93, 1.09)	0.91 (0.46, 1.57)	1.05 (0.93, 1.14)	0.91 (0.68, 1.18)	1.02 (0.97, 1.06)
Abnormal Spirometry	0.99 (0.69, 1.38)	1.04 (0.97, 1.11)	1.16 (0.69, 1.82)	*1.08 (0.98, 1.17)*	1.01 (0.81, 1.25)	1.03 (0.99, 1.07)
[a]Spirometry Obstruction	0.66 (0.36, 1.06)	0.97 (0.85, 1.06)	0.62 (0.26, 1.27)	0.94 (0.77, 1.09)	0.79 (0.55, 1.06)	0.98 (0.90 1.04)
Restriction + Mixed	**1.88 (1.10, 3.34)**	**1.18 (1.06, 1.36)**	**2.26 (1.23, 4.34)**	**1.26 (1.10, 1.52)**	**1.49 (1.08, 2.07)**	**1.11 (1.04, 1.21)**
[b]Abnormal IOS	1.00 (0.79, 1.27)	1.02 (0.98, 1.05)	1.08 (0.74, 1.54)	*1.06 (1.00, 1.13)*	1.02 (0.88, 1.19)	*1.02 (1.00, 1.05)*
[a,b]IOS Large Airways	1.13 (0.78, 1.62)	1.01 (0.95, 1.06)	1.24 (0.68, 2.08)	1.06 (0.96, 1.15)	1.10 (0.87, 1.38)	1.02 (0.98, 1.06)
Small + Small and Large Airways	1.00 (0.77, 1.29)	*1.02 (0.98, 1.06)*	1.07 (0.70, 1.58)	*1.07 (0.99, 1.15)*	1.02 (0.86, 1.20)	*1.03 (1.00, 1.06)*

Covariates: Age, BMI, Height, Tenure, Sex, Smoke, Race, Allergic status; CE= cumulative exposure; **Bold = p < 0.05**; *Italics = 0.05 < p < 0.10*; Estimates of slope and their 95% CI are expressed as percentage points per 10 ppb; Estimates of OR and their 95% CI are

expressed as odds per 10 ppb; Models modified to address quasi or complete separation, all logistic and polytomous models used binary race, whites vs. non-whites; a= sex excluded; b= height included; c= p-value was < 0.05, Wald confidence interval did not include 1, however profile likelihood confidence interval included 1.

Supplementary Table S2: Effects of covariates on outcome variables in overall models of lung function with metrics of exposure to diacetyl and 2,3-pentanedione and their sum.

	Age			BMI			Tenure			Height			Sex (F)			Ever Smoke (Y)			Race (Hispanic)			Race (Other)			Allergic Status (Y)		
**Diacetyl**	**P95**	**Ave**	**CE**	**P95**	**Ave**	**CE**	**P95**	**Ave**	**CE**	**P95**	**Ave**	**CE**	**P95**	**Ave**	**CE**	**P95**	**Ave**	**CE**	**P95**	**Ave**	**CE**	**P95**	**Ave**	**CE**	**P95**	**Ave**	**CE**
	**β**	**β**	**β**	**β**	**β**	**β**	**β**	**β**	**β**	**β**	**β**	**β**	**β**	**β**	**β**	**β**	**β**	**β**	**β**	**β**	**β**	**β**	**β**	**β**	**β**	**β**	**β**
$ppFEV_1$	-0.09	-0.09	-0.09	-0.22^{+}	-0.21^{+}	-0.21^{+}	0.29*	0.27^{+}	0.25	-	-	-	3.55*	3.90*	3.87*	-0.90	-0.90	-0.90	2.79	1.95	2.04	2.78	2.18	2.22	0.76	0.81	0.79
ppFVC	0.00	0.00	0.00	-0.25*	-0.24*	-0.23*	0.06	0.02	0.14	-	-	-	2.69*	2.78*	2.82*	0.94	0.94	0.95	0.03	0.06	-0.14	0.75	0.59	0.49	0.27	0.10	0.16
$ppFEV_1/FVC$	-0.10*	-0.10*	-0.09*	0.00	0.00	0.00	0.20*	0.23*	0.10	-	-	-	0.60	0.88	0.80	-2.10*	-2.09*	-2.10*	2.44*	1.54	1.86	2.22	1.75	1.91	0.50	0.72	0.62
	**OR**	**OR**	**OR**	**OR**	**OR**	**OR**	**OR**	**OR**	**OR**	**OR**	**OR**	**OR**	**OR**	**OR**	**OR**	**OR**	**OR**	**OR**	**OR**	**OR**	**OR**	**OR**	**OR**	**OR**	**OR**	**OR**	**OR**
FEV1 < LLN	1.02	1.02	1.03	1.08^{+}	1.07	1.07	0.95	0.97	0.92	-	-	-	0.31	0.27^{+}	0.27^{+}	3.92*	3.59*	3.61*	-	-	-	0.49	0.61	0.66	0.68	0.77	0.72
FVC < LLN	1.00	1.00	1.01	1.12*	1.10*	1.09*	0.86	0.96	0.70*	-	-	-	-	-	-	2.28	2.11	1.97	-	-	-	0.58	0.64	0.79	0.76	1.19	1.04
Abnorm. Spiro.	1.02	1.02	1.02	1.02	1.01	1.01	0.90*	0.91*	0.88*	-	-	-	0.68	0.59	0.63	1.64	1.63	1.63	-	-	-	0.47^{+}	0.63	0.65	1.15	1.15	1.20
Obstruction<br>Restriction	1.03<br>1.01	1.03<br>1.00	1.03<br>1.01	0.97<br>1.12*	0.96<br>1.10*	0.97<br>1.09^{+}	0.92^{+}<br>0.86^{+}	0.90*<br>0.95	0.94<br>0.70*	-<br>-	-<br>-	-<br>-	-<br>-	-<br>-	-<br>-	1.68<br>2.37	1.76<br>2.16	1.69<br>2.04	-<br>-	-<br>-	-<br>-	0.42<br>0.54	0.61<br>0.62	0.47<br>0.74	1.20<br>1.78	1.07<br>1.20	1.17<br>1.05
Abnormal IOS	0.99	0.99	0.99	1.15*	1.14*	1.14*	0.97	0.98	0.96	0.82*	0.82*	0.82*	0.69	0.63	0.66	0.93	0.93	0.94	-	-	-	0.88	1.00	0.96	0.82	0.81	0.81
Large Airways<br>Small + S & L	1.00<br>0.99	0.99<br>0.99	1.00<br>0.99	1.10*<br>1.17*	1.10*<br>1.16*	1.10*<br>1.16*	0.99<br>0.95	1.00<br>0.97	0.98<br>0.94	0.93<br>0.82*	0.93<br>0.83*	0.93<br>0.83*	-<br>-	-<br>-	-<br>-	0.76<br>1.05	0.76<br>1.03	0.76<br>1.04	-<br>-	-<br>-	-<br>-	0.81<br>1.04	0.71<br>1.31	0.78<br>1.21	0.55<br>0.97	0.57<br>0.94	0.55<br>0.95
**2,3-Pentanedione**	**β**	**β**	**β**	**β**	**β**	**β**	**β**	**β**	**β**	**β**	**β**	**β**	**β**	**β**	**β**	**β**	**β**	**β**	**β**	**β**	**β**	**β**	**β**	**β**	**β**	**β**	**β**
$ppFEV_1$	-0.10	-0.09	-0.10	-0.22^{+}	-0.21^{+}	-0.20^{+}	0.31*	0.27^{+}	-0.36*	-	-	-	3.74*	3.84*	3.80*	-0.88	-0.89	-0.86	2.83^{+}	2.17	2.46	2.58	2.28	2.35	0.85	0.75	0.65
ppFVC	-0.00	-0.00	-0.01	-0.25*	-0.24*	-0.23*	0.07	0.02	0.21	-	-	-	2.86*	2.81*	2.86*	0.96	1.02	1.01	0.03	0.10	0.08	0.56	0.49	0.46	0.35	0.08	0.05
$ppFEV_1/FVC$	-0.10*	-0.10*	-0.09*	0.00	0.00	0.00	0.21*	0.22*	0.14	-	-	-	0.63	0.77	0.69	-2.09*	-2.16*	-2.12*	2.48*	1.76	2.08^{+}	2.20	1.95	2.06	0.53	0.67	0.59
	**OR**	**OR**	**OR**	**OR**	**OR**	**OR**	**OR**	**OR**	**OR**	**OR**	**OR**	**OR**	**OR**	**OR**	**OR**	**OR**	**OR**	**OR**	**OR**	**OR**	**OR**	**OR**	**OR**	**OR**	**OR**	**OR**	**OR**
FEV1 < LLN	1.03	1.02	1.03	1.08^{+}	1.07^{+}	1.07	0.95	0.99	0.90	-	-	-	0.24^{+}	0.25^{+}	0.26^{+}	4.05*	3.39*	3.64*	-	-	-	0.49	0.52	0.58	0.72	0.84	0.81
FVC < LLN	1.01	1.00	1.01	1.12*	1.11*	1.10*	0.87	0.95	069*	-	-	-	-	-	-	2.00	1.76	1.76	-	-	-	0.83	0.87	0.74	0.69	0.90	0.98
Abnorm. Spiro.	1.02	1.02	1.02	1.02	1.01	1.01	0.90*	0.92^{+}	0.87*	-	-	-	0.60	0.60	0.61	1.63	1.61	1.61	-	-	-	0.50	0.58	0.54	1.14	1.20	1.24
Obstruction<br>Restriction	1.03<br>1.01	1.03<br>1.01	1.03<br>1.01	0.97<br>1.11*	0.97<br>1.10*	0.97<br>1.10*	0.91^{+}<br>0.87	0.90^{+}<br>0.94	0.94<br>0.69*	-<br>-	-<br>-	-<br>-	-<br>-	-<br>-	-<br>-	1.67<br>2.10	1.75<br>1.82	1.70<br>1.82	-<br>-	-<br>-	-<br>-	0.39^{+}<br>0.76	0.52<br>0.84	0.46<br>0.70	1.18<br>0.71	1.09<br>0.91	1.17<br>1.00
Abnormal IOS	0.99	0.99	0.99	1.15*	1.14*	1.14*	0.97	0.98	0.94	0.82*	0.83*	0.83*	0.65	0.65	0.66	0.92	0.92	0.91	-	-	-	0.94	0.97	0.93	0.81	0.83	0.85
Large Airways<br>Small + S & L	0.99<br>0.99	0.99<br>0.99	1.00<br>0.99	1.10*<br>1.17*	1.10*<br>1.16*	1.10*<br>1.16*	1.00<br>0.96	1.00<br>0.97	0.96<br>0.92	0.93<br>0.82*	0.93<br>0.83*	0.93<br>0.83*	-<br>-	-<br>-	-<br>-	0.76<br>1.02	0.74<br>1.02	0.75<br>1.01	-<br>-	-<br>-	-<br>-	0.83<br>1.17	0.73<br>1.26	0.75<br>1.18	0.55<br>0.94	0.57<br>0.96	0.57<br>0.99
**Sum_{DA+PD}**	**β**	**β**	**β**	**β**	**β**	**β**	**β**	**β**	**β**	**β**	**β**	**β**	**β**	**β**	**β**	**β**	**β**	**β**	**β**	**β**	**β**	**β**	**β**	**β**	**β**	**β**	**β**
$ppFEV_1$	-0.10	-0.09	-0.10	-0.22^{+}	-0.21^{+}	-0.21^{+}	0.31*	0.27^{+}	0.32^{+}	-	-	-	3.64*	3.86*	3.79*	-0.90	-0.90	-0.89	2.95^{+}	2.10	2.36	2.70	2.25	2.30	0.79	0.77	0.69
ppFVC	-0.00	-0.00	-0.01	-0.25*	-0.24*	-0.23*	0.07	0.02	0.19	-	-	-	2.77*	2.78*	2.81*	0.94	0.97	0.97	0.13	0.13	0.06	0.66	0.57	0.54	0.29	0.07	0.07
$ppFEV_1/FVC$	-0.10*	-0.10*	-0.09*	0.00	0.00	0.00	0.21*	0.22*	0.12	-	-	-	0.61	0.82	0.73	-2.10*	-2.12*	-2.11*	2.51*	1.65	1.98^{+}	2.23	1.85	1.99	0.51	0.70	0.61
	**OR**	**OR**	**OR**	**OR**	**OR**	**OR**	**OR**	**OR**	**OR**	**OR**	**OR**	**OR**	**OR**	**OR**	**OR**	**OR**	**OR**	**OR**	**OR**	**OR**	**OR**	**OR**	**OR**	**OR**	**OR**	**OR**	**OR**
FEV1 < LLN	1.03	1.02	1.03	1.08^{+}	1.07	1.06	0.95	0.98	0.91	-	-	-	0.28	0.28	0.27^{+}	4.15*	3.56*	3.66*	-	-	-	0.46	0.52	0.61	0.75	0.85	0.79
FVC < LLN	1.01	1.00	1.01	1.12*	1.10*	1.10*	0.86	0.96	0.70*	-	-	-	-	-	-	2.14	1.95	1.87	-	-	-	0.69	0.68	0.75	0.76	1.16	1.08
Abnorm. Spiro.	1.02	1.02	1.02	1.02	1.01	1.01	0.90*	0.92^{+}	0.87*	-	-	-	0.63	0.60	0.62	1.66	1.63	1.63	-	-	-	0.47^{+}	0.61	0.54	1.16	1.17	1.24
Obstruction<br>Restriction	1.03<br>1.01	1.03<br>1.00	1.03<br>1.01	0.97<br>1.12*	0.97<br>1.10*	0.97<br>1.09*	0.91^{+}<br>0.85	0.90^{+}<br>0.95	0.94<br>0.69*	-<br>-	-<br>-	-<br>-	-<br>-	-<br>-	-<br>-	1.66<br>2.23	1.76<br>2.00	1.69<br>1.94	-<br>-	-<br>-	-<br>-	0.39^{+}<br>0.64	0.57<br>0.66	0.46<br>0.71	1.19<br>0.78	1.07<br>1.17	1.17<br>1.10
Abnormal IOS	0.99	0.99	0.99	1.15*	1.14*	1.14*	0.97	0.98	0.94^{+}	0.82*	0.82*	0.83*	0.67	0.65	0.68	0.93	0.93	0.93	-	-	-	0.91	0.97	0.91	0.82	0.82	0.85
Large Airways<br>Small + S & L	0.99<br>0.99	0.99<br>0.99	1.00<br>0.99	1.10*<br>1.17*	1.10*<br>1.16*	1.10*<br>1.16*	0.99<br>0.96	1.00<br>0.97	0.96<br>0.92^{+}	0.93<br>0.82*	0.93<br>0.83*	0.93<br>0.83*	-<br>-	-<br>-	-<br>-	0.76<br>1.03	0.75<br>1.03	0.76<br>1.03	-<br>-	-<br>-	-<br>-	0.81<br>1.11	0.69<br>1.27	0.74<br>1.14	0.55<br>0.96	0.58<br>0.95	0.56<br>1.00

[*] $=p<0.05$; [+]$=0.05<p<0.1$; [-] = variable not included in the model; BMI = body mass index; Ave = average exposure; CE= cumulative exposure; β = betas; OR = odds ratio; Reference for: Sex=male, Smoke=Never Smoker, Race=White, Allergic Status=No.

Supplementary Table S3: Fit statistics for associations of lung function with metrics of worklife exposure to diacetyl, 2,3-pentanedione and their sum

Health Outcome	Diacetyl			2,3-Pentanedione			Sum_{DA+PD}		
	Highest P95 β/SE (AIC)	Average β/SE (AIC)	CE β/SE (AIC)	Highest P95 β/SE (AIC)	Average β/SE (AIC)	CE β/SE (AIC)	Highest P95 β/SE (AIC)	Average β/SE (AIC)	CE β/SE (AIC)
$ppFEV_1$	-1.50 (2947.4)	0.21 (2949.6)	0.16 (2949.6)	-2.15 (2945.1)	-0.07 (2949.6)	-0.81 (2949.0)	-2.03 (2945.5)	0.03 (2949.6)	-0.49 (2949.4)
ppFVC	-1.43 (2889.4)	-0.81 (2890.8)	-0.94 (2890.6)	-1.91 (2887.8)	-1.05 (2890.3)	-1.65 (2888.7)	-1.82 (2888.1)	-0.92 (2890.6)	-1.40 (2889.5)
$ppFEV_1$/FVC	-0.46 (2627.4)	1.44 (2625.5)	1.43 (2625.5)	-0.83(2626.9)	1.26 (2626.0)	0.86 (2626.9)	-0.77 (2627.0)	1.28 (2626.0)	1.06 (2626.5)
PP FEF_{25-75}	-0.64 (3533.1)	0.60 (3533.2)	1.80 (3530.3)	-1.30 (3531.8)	0.28 (3533.4)	0.91 (3532.7)	-1.15 (3532.2)	0.32 (3533.4)	1.16 (3532.2)
	Highest P95 β/SE (AIC)	Average β/SE (AIC)	CE β/SE (AIC)	Highest P95 β/SE (AIC)	Average β/SE (AIC)	CE β/SE (AIC)	Highest P95 β/SE (AIC)	Average β/SE (AIC)	CE β/SE (AIC)
FEV_1 < LLN	2.29 (124.0)	0.76 (127.7)	0.99 (127.4)	3.09 (120.8)	2.17 (124.4)	2.05 (124.9)	2.90 (121.5)	1.42 (126.4)	1.57 (126.2)
FVC < LLN	3.68 (95.5)	2.35 (102.1)	2.89 (98.5)	3.44 (98.5)	2.77 (101.1)	2.88 (96.7)	3.47 (97.5)	2.50 (101.7)	2.88 (97.4)
Abnormal Spirometry	2.35 (241.6)	-0.08 (246.3)	1.13 (245.1)	2.85 (239.6)	0.06 (245.9)	1.78 (243.6)	2.80 (239.9)	0.12 (246.2)	1.51 (244.3)
Obstruction	0.05 (286.9)	-1.56 (290.6)	-0.57 (289.5)	0.99 (289.1)	-1.19 (290.9)	-0.63 (287.7)	0.73 (288.5)	-1.44 (290.7)	-0.60 (288.4)
Restriction + Mixed	3.68 (NA)	2.27 (NA)	2.87 (NA)	3.50 (NA)	2.68 (NA)	2.86 (NA)	3.50 (NA)	2.42 (NA)	2.85 (NA)
Abnormal IOS	1.93 (389.6)	0.02 (393.2)	0.87 (392.5)	1.78 (390.2)	0.39 (393.1)	1.85 (389.8)	1.94 (389.6)	0.27 (393.2)	1.73 (390.3)
Small + Small & Large Airways	2.58 (516.3)	-0.01 (522.4)	1.10 (521.7)	2.29 (517.6)	0.31 (522.4)	1.80 (519.0)	2.45 (517.0)	0.19 (522.3)	1.77 (519.3)
Large Airways	0.03 (NA)	0.68 (NA)	0.47 (NA)	-0.21 (NA)	0.76 (NA)	1.26 (NA)	0.06 (NA)	0.83 (NA)	1.12 (NA)

Negative sign indicates negative coefficient; β/SE is a measure of the precision of the parameter estimate (larger is better); CE = cumulative exposure; NA =Not applicable, AIC=Akaike information criterion, model fit value is for the whole model (smaller is better).

Supplementary Table S4: Associations of lung function with metrics of worklife exposure to diacetyl, 2,3-pentanedione, and their sum for the subset of flavoring workers (n=71)

Health Outcome	Diacetyl			2,3-Pentanedione			Sum$_{DA+PD}$		
	Highest P95 Slope (95% CI)	Average Slope (95% CI)	CE Slope (95% CI)	Highest P95 Slope (95% CI)	Average Slope (95% CI)	CE Slope (95% CI)	Highest P95 Slope (95% CI)	Average Slope (95% CI)	CE Slope (95% CI)
ppFEV$_1$	**-0.53 (-0.97, -0.10)**	0.15 (-3.69, 3.99)	-0.31 (-0.85, 0.23)	**-0.36 (-0.70, -0.03)**	0.27 (-3.49, 4.02)	-0.45 (-1.02, 0.13)	**-0.23 (-0.42, -0.04)**	0.03 (-1.97, 2.04)	-0.20 (-0.47, 0.08)
ppFVC	**-0.46 (-0.88, -0.04)**	-2.30 (-5.92, 1.32)	-0.33 (-0.84, 0.18)	*-0.30 (-0.62, 0.01)*	-1.53 (-5.09, 2.03)	*-0.51 (-1.05, 0.03)*	**-0.19 (-0.38, -0.01)**	-1.10 (-2.99, 0.79)	-0.21 (-0.48, 0.05)
ppFEV$_1$/FVC	-0.12 (-0.43, 0.19)	1.88 (-0.71, 4.48)	-0.05 (-0.42, 0.32)	-0.10 (-0.33, 0.14)	1.24 (-1.32, 3.79)	-0.02 (-0.42, 0.38)	-0.06 (-0.19, 0.08)	0.81 (-0.54, 2.17)	-0.02 (-0.21, 0.17)
	Highest P95 OR (95% CI)	Average OR (95% CI)	CE OR (95% CI)	Highest P95 OR (95% CI)	Average OR (95% CI)	CE OR (95% CI)	Highest P95 OR (95% CI)	Average OR (95% CI)	CE OR (95% CI)
[a]FEV$_1$ < LLN	**1.21 (1.04, 1.58)**	*5.59 (1.32, 91.26)*	*1.39 (1.05, 2.27)*	**1.14 (1.03, 1.33)**	*2.29 (0.81, 7.24)*	*1.32 (1.02, 2.06)*	**1.09 (1.02, 1.22)**	*2.24 (1.11, 6.53)*	*1.18 (1.02, 1.51)*
[a]FVC < LLN	*1.26 (1.03, 1.83)*	[c,e]*1.36 (1.09, 2.32)*	[d]*1.99 (1.22, 7.60)*	*1.12 (1.01, 1.33)*	*4.08 (1.17, 31.93)*	[d]*2.44 (1.30, 11.48)*	*1.09 (1.01, 1.26)*	[e]1.753 (1.085, 19.31)	[d]*1.50 (1.13, 3.38)*
[a]FEV$_1$/FVC < LLN	1.07 (0.96, 1.20)	0.77 (0.30, 1.72)	1.06 (0.93, 1.21)	1.05 (0.97, 1.13)	0.84 (0.26, 1.85)	1.06 (0.92, 1.21)	1.03 (0.99, 1.08)	0.91 (0.55, 1.39)	1.03 (0.96, 1.10)
[a]Abnormal Spirometry	*1.11 (1.00, 1.25)*	1.05 (0.48, 2.26)	1.08 (0.95, 1.24	*1.07 (1.00, 1.15)*	0.98 (0.37, 1.98)	1.07 (0.92, 1.23)	**1.05 (1.00, 1.10)**	1.03 (0.67, 1.53)	1.04 (0.97, 1.11)
[a]Spirometry Obstruction	1.05 (0.91, 1.20)	[c]0.43 (0.09, 1.09)	[d]0.94 (0.72, 1.11)	1.04 (0.93, 1.13)	[d]0.22 (0.02, 0.94)	[d]0.84 (0.55, 1.09)	1.03 (0.96, 1.08)	0.54 (0.18, 1.06)	[d]0.95 (0.80, 1.05)
Restriction + Mixed	*1.27 (1.03, 1.86)*	[c,e]*1.36 (1.09, 2.31)*	[d]*1.99 (1.22, 7.60)*	*1.13 (1.01, 1.34)*	[d]*3.14 (1.07, 17.17)*	[d]*2.43 (1.30, 11.39)*	*1.09 (1.01, 1.26)*	[e]1.74 (1.08, 18.81)	[d]*1.50 (1.13, 3.38)*
[b]Abnormal IOS	*1.09 (1.00, 1.19)*	1.30 (0.68, 2.51)	1.04 (0.94, 1.15)	*1.05 (0.99, 1.13)*	1.17 (0.61, 2.25)	1.06 (0.96, 1.18)	*1.04 (1.00, 1.08)*	1.14 (0.81, 1.62)	1.03 (0.98, 1.08)
[a,b,d]IOS Large Airways	0.92 (0.63, 1.09)	0.76 (0.25, 1.76)	0.97 (0.81, 1.11)	0.94 (0.65, 1.07)	0.92 (0.25, 2.34)	1.02 (0.85, 1.18)	0.97 (0.80, 1.04)	0.91 (0.50, 1.46)	1.00 (0.91, 1.07)
Small + Small & Large Airways	**1.13 (1.03, 1.28)**	1.43 (0.79, 2.69)	1.08 (0.97, 1.22)	**1.08 (1.01, 1.17)**	1.27 (0.63, 2.54)	1.09 (0.98, 1.25)	**1.06 (1.01, 1.11)**	1.21 (0.86, 1.75)	1.04 (0.99, 1.11)

Covariates: Age, BMI, Tenure, Sex, Smoke, Race, Allergic status; CE = cumulative exposure; **Bold = p < 0.05**; *Italics = 0.05 < p < 0.10*; Estimates of slope and their 95% CI are expressed as percentage points per 10 ppb; Estimates of OR and their 95% CI are expressed as odds per 10 ppb; Models modified to address quasi or complete separation, all logistic and polytomous models used binary race, whites vs. non-whites; a= sex excluded; b= height included, c=smoke, race, and allergic status excluded; d=race and allergic status excluded, e=estimate is per ppb.

Supplementary Table S5: Associations of lung function with metrics of worklife exposure to diacetyl, 2,3-pentanedione and their sum for a subset of non-flavoring workers (n=313)

Health Outcome	Diacetyl			2,3-Pentanedione			Sum		
	Highest P95 Slope (95% CI)	Average Slope (95% CI)	CE Slope (95% CI)	Highest P95 Slope (95% CI)	Average Slope (95% CI)	CE Slope (95% CI)	Highest P95 Slope (95% CI)	Average Slope (95% CI)	CE Slope (95% CI)
$ppFEV_1$	0.31 (-0.22, 0.84)	0.10 (-1.34, 1.55)	0.10 (-0.20, 0.39)	0.56 (-0.77, 1.88)	-0.24 (-2.90, 2.41)	0.002 (-0.54, 0.55)	0.30 (-0.20, 0.79)	-0.01 (-0.98, 0.95)	0.02 (-0.19, 0.22)
ppFVC	0.18 (-0.30, 0.67)	-0.31 (-1.63, 1.02)	-0.09 (-0.36, 0.18)	0.11 (-1.11, 1.32)	-0.99 (-3.42, 1.45)	-0.31 (-0.82, 0.19)	0.15 (-0.31, 0.60)	-0.25 (-1.13, 0.64)	-0.10 (-0.29, 0.09)
$ppFEV_1/FVC$	0.14 (-0.20, 0.48)	0.47 (-0.45, 1.39)	**0.19 (0.00, 0.38)**	0.48 (-0.37, 1.32)	0.90 (-0.79, 2.59)	*0.32 (-0.02, 0.67)*	0.16 (-0.15, 0.47)	0.28 (-0.34, 0.89)	*0.12 (-0.01, 0.25)*
	Highest P95 OR (95% CI)	Average OR (95% CI)	CE OR (95% CI)	Highest P95 OR (95% CI)	Average OR (95% CI)	CE OR (95% CI)	Highest P95 OR (95% CI)	Average OR (95% CI)	CE OR (95% CI)
FEV_1 < LLN	0.95 (0.69, 1.17)	1.06 (0.47, 2.07)	1.00 (0.88, 1.11)	1.31 (0.74, 2.30)	2.55 (0.80, 8.04)	1.11 (0.89, 1.37)	1.00 (0.79, 1.24)	1.19 (0.73, 1.87)	1.02 (0.93, 1.10)
[a]FVC < LLN	**1.23 (1.01, 1.47)**	1.66 (0.88, 3.34)	**1.19 (1.02, 1.44)**	**2.68 (1.20, 7.94)**	**3.79 (1.15, 15.6)**	**1.65 (1.21, 2.56)**	*1.30 (1.00, 1.77)*	1.37 (0.90, 2.18)	**1.15 (1.03, 1.31)**
FEV_1/FVC < LLN	0.87 (0.64, 1.07)	0.80 (0.41, 1.37)	0.98 (0.85, 1.09)	0.91 (0.57, 1.39)	0.88 (0.32, 2.13)	0.98 (0.76, 1.20)	0.92 (0.76, 1.09)	0.90 (0.60, 1.28)	0.99 (0.90, 1.07)
Abnormal Spirometry	1.01 (0.85, 1.15)	1.04 (0.67, 1.56)	1.03 (0.94, 1.12)	1.09 (0.74, 1.59)	1.43 (0.68, 2.92)	1.13 (0.96, 1.33)	0.99 (0.85, 1.14)	1.05 (0.78, 1.37)	1.03 (0.97, 1.10)
[c]Spirometry Obstruction	0.84 (0.62, 1.05)	0.70 (0.37, 1.15)	0.95 (0.80, 1.07)	0.87 (0.55, 1.33)	0.74 (0.28, 1.70)	0.94 (0.72, 1.16)	0.90 (0.74, 1.06)	0.83 (0.56, 1.13)	0.97 (0.87, 1.05)
Restriction + Mixed	*1.18 (0.97, 1.41)*	1.35 (0.76, 2.44)	*1.15 (0.99, 1.36)*	**2.35 (1.10, 6.37)**	*2.83 (0.92, 10.24)*	**1.56 (1.17, 2.31)**	1.21 (0.95, 1.57)	1.22 (0.83, 1.82)	**1.12 (1.01, 1.25)**
[b]Abnormal IOS	1.03 (0.93, 1.14)	0.95 (0.73, 1.23)	1.01 (0.97, 1.05)	0.94 (0.72, 1.22)	0.91 (0.55, 1.48)	1.05 (0.95, 1.17)	1.01 (0.92, 1.11)	0.97 (0.81, 1.16)	1.02 (0.98, 1.06)
[d,b]IOS Large Airways	1.01 (0.85, 1.17)	1.08 (0.72, 1.59)	1.01 (0.94, 1.06)	0.98 (0.65, 1.47)	1.13 (0.52, 2.38)	1.08 (0.93, 1.25)	1.03 (0.89, 1.19)	1.06 (0.81, 1.38)	1.03 (0.97, 1.08)
Small + Small & Large Airways	1.05 (0.94, 1.17)	0.93 (0.69, 1.24)	1.01 (0.96, 1.06)	0.95 (0.71, 1.27)	0.87 (0.50, 1.48)	1.05 (0.92, 1.18)	1.01 (0.91, 1.12)	0.95 (0.78, 1.16)	1.02 (0.98, 1.07)

Covariates: Age, BMI, Tenure, Sex, Smoke, Race, Allergic status; CE = cumulative exposure; **Bold = p < 0.05**; *Italics = 0.05 < p < 0.10*; Estimates of slope and their 95% CI are expressed as percentage points per 10 ppb; Estimates of OR and their 95% CI are expressed as odds per 10 ppb; Models modified to address quasi or complete separation, all logistic and polytomous models used binary race, whites vs. non-whites; a= sex excluded; b= height included; c=sex, smoke, race excluded; d=sex and smoke excluded.

1.3 References

1. Bickel S, Popler J, Lesnick B, Eid N. Impulse oscillometry: interpretation and practical applications. Chest. 2014;146(3):841-7.
2. Berger KI, Reibman J, Oppenheimer BW, Vlahos I, Harrison D, Goldring RM. Lessons from the World Trade Center disaster: airway disease presenting as restrictive dysfunction. Chest. 2013;144(1):249-57.
3. Jordan HT, Friedman SM, Reibman J, Goldring RM, Miller Archie SA, Ortega F, et al. Risk factors for persistence of lower respiratory symptoms among community members exposed to the 2001 World Trade Center terrorist attacks. Occup Environ Med. 2017;74(6):449-55.
4. King GG, Bates J, Berger KI, Calverley P, de Melo PL, Dellacà RL, et al. Technical standards for respiratory oscillometry. Eur Respir J. 2020;55(2).
5. Cottini M, Licini A, Lombardi C, Bagnasco D, Comberiati P, Berti A. Small airway dysfunction and poor asthma control: a dangerous liaison. Clin Mol Allergy. 2021;19(1):7.
6. Berger KI, Wohlleber M, Goldring RM, Reibman J, Farfel MR, Friedman SM, et al. Respiratory impedance measured using impulse oscillometry in a healthy urban population. ERJ Open Res. 2021;7(1).
7. J. SH. Questions about impulse oscillometry. In: Bailey R, editor. 2015.
8. Vogel J, U S. Impulse oscillometry: Analysis of lung mechanics in general practice and clinic, epidemiological and experimental research. Frankfurt: PMI-Verlagsgruppe; 1994.
9. LeBouf RF, Blackley BH, Fortner AR, Stanton M, Martin SB, Groth CP, et al. Exposures and Emissions in Coffee Roasting Facilities and Cafés: Diacetyl, 2,3-Pentanedione, and Other Volatile Organic Compounds. Frontiers in public health. 2020;8:561740.
10. Bullock WH, Ignacio JS. A strategy for assessing and managing occupational exposures: AIHA; 2006.
11. Pulido JA, Barrero LH, Mathiassen SE, Dennerlein JT. Correctness of Self-Reported Task Durations: A Systematic Review. Ann Work Expo Health. 2017;62(1):1-16.
12. Smith TJ. Occupational exposure and dose over time: limitations of cumulative exposure. American journal of industrial medicine. 1992;21(1):35-51.
